# Covalent α-Synuclein Dimers: Chemico-Physical and Aggregation Properties

**DOI:** 10.1371/journal.pone.0050027

**Published:** 2012-12-13

**Authors:** Micaela Pivato, Giorgia De Franceschi, Laura Tosatto, Erica Frare, Dhruv Kumar, Daniel Aioanei, Marco Brucale, Isabella Tessari, Marco Bisaglia, Bruno Samori, Patrizia Polverino de Laureto, Luigi Bubacco

**Affiliations:** 1 CRIBI Biotechnology Centre, University of Padova, Padova, Italy; 2 University of Padova, Department of Biology, Padova, Italy; 3 University of Bologna, Department of Biochemistry, Bologna, Italy; 4 CNR, Institute of Nanostructured Materials (ISMN), Montelibretti, Roma, Italy; University of South Florida College of Medicine, United States of America

## Abstract

The aggregation of α-synuclein into amyloid fibrils constitutes a key step in the onset of Parkinson's disease. Amyloid fibrils of α-synuclein are the major component of Lewy bodies, histological hallmarks of the disease. Little is known about the mechanism of aggregation of α-synuclein. During this process, α-synuclein forms transient intermediates that are considered to be toxic species. The dimerization of α-synuclein could represent a rate-limiting step in the aggregation of the protein. Here, we analyzed four covalent dimers of α-synuclein, obtained by covalent link of the N-terms, C-terms, tandem cloning of two sequences and tandem juxtaposition in one protein of the 1–104 and 29–140 sequences. Their biophysical properties in solution were determined by CD, FT-IR and NMR spectroscopies. SDS-induced folding was also studied. The fibrils formation was analyzed by ThT and polarization fluorescence assays. Their morphology was investigated by TEM and AFM-based quantitative morphometric analysis. All dimers were found to be devoid of ordered secondary structure under physiological conditions and undergo α-helical transition upon interaction with SDS. All protein species are able to form amyloid-like fibrils. The reciprocal orientation of the α-synuclein monomers in the dimeric constructs affects the kinetics of the aggregation process and a scale of relative amyloidogenic propensity was determined. Structural investigations by FT IR spectroscopy, and proteolytic mapping of the fibril core did not evidence remarkable difference among the species, whereas morphological analyses showed that fibrils formed by dimers display a lower and diversified level of organization in comparison with α-synuclein fibrils. This study demonstrates that although α-synuclein dimerization does not imply the acquisition of a preferred conformation by the participating monomers, it can strongly affect the aggregation properties of the molecules. The results presented highlight a substantial role of the relative orientation of the individual monomer in the definition of the fibril higher structural levels.

## Introduction

α-Synuclein (aS) is a small (140 amino acids) protein, highly expressed in the central nervous system where it constitutes about 0.5–1.0% of the entire cytosolic protein content. In fibrillar form, it is the major component of intracellular deposits of proteins and lipids called Lewy bodies and Lewy neurites, which are hallmarks of Parkinson's disease (PD) and other related neurological disorders [1], [2]. These inclusions share common structural features, including a high β-sheet content and a typical cross-β X-ray diffraction pattern [3]. Point mutations or multiplication of the aS gene have been associated to early-onset autosomal dominant PD [4]–[8]. Although the mechanisms by which the aS exerts its toxicity are not clarified yet, strong evidences support the direct link between the aggregation of aS and the degeneration of the dopaminergic neurons in PD [9].

The amino acidic sequence of aS includes a conserved N-terminal domain containing a series of amphipathic repeats that modulates membrane binding; a central region, between residues 61–95, that comprises the highly aggregation-prone NAC sequence [10], [11]; a C-terminal region enriched in acidic residues and prolines. aS lacks of ordered secondary structure under physiological conditions, as deduced by far-UV circular dichroism (CD) and Fourier transform IR (FT-IR) measurements [12]. For this reason, it has been widely accepted that aS is an intrinsically disordered protein [13], [14]. Nuclear magnetic resonance (NMR) and small angle X-ray scattering (SAXS) studies evidenced that aS exists as an ensemble of dynamic structures that could be, on average, more compact than a random coil [15]–[18]. Recently, it was proposed that aS is *in vivo* a tetramer with α-helical structure [19], [20]. This proposal was questioned by other studies [21]. The clarification of aS physiological conformation remains fundamental to the understanding of its protein function.

The aggregation of aS could be described as a process, which follows a nucleated polymerization model. Unfolded aS molecules are initially distributed among several related conformations. At the onset of the fibrillization process, soluble species undergo self-assembly into stable oligomeric intermediates able to grow through monomer accretion, thereby evolving into final amyloid-like fibrils [15], [22], [23]. The presence of small oligomers, ranging in size from dimers to few monomers, during aS fibril formation *in vitro* has been very recently demonstrated by FRET. Furthermore, several types of oligomers, with different degrees of toxicity have been described [24].

aS stable covalent dimers were obtained through oxidation or nitration mechanisms or via di-tyrosine binding [25]–[27]. These studies provided relevant evidence of the implication of oxidative stress in the onset of PD, however, the mechanism whereby aS polymerizes in living cells remains incompletely understood. The formation of aS dimers could be regarded as the rate-limiting step for the aggregation, since dimer formation could lock some unfolded conformation, shifting the equilibrium away from the native state.

To elucidate the role of dimerization in the aS aggregation process, we produced and characterized four dimers of aS, obtained by covalently linking two aS monomers via their terminals. A cysteine residue was added at either the N- or C-terminal of aS, providing head-to-head “NN” or tail-to-tail “CC” dimerization through the formation of a disulfide bond. An “NC” dimer, formed by a tandem repeat of the aS sequence, was obtained as a single polypeptide chain. Another dimer, called “double core” or “DC” dimer, is constituted by two consecutive central, highly amyloidogenic regions, containing aS residues from 1 to 104 and from 29 to 140. This last dimer was conceived to both draw up the hydrophobic and amyloidogenic regions, avoiding the interferences of the terminal parts, and to impose a constrain of proximity between the two amyloidogenic regions of aS.

Our study demonstrates that bringing aS molecules in close proximity is not a sufficient condition for the protein to assume a specific conformation. On the other hand, the relative orientation of each molecule in the dimer plays a critical role in the structural arrangement of aS fibrils.

## Materials and Methods

Proteinase K from *Tritirachium album and* porcine trypsin were obtained from Sigma Chem. Co., St. Louis, MO. All other chemicals were of analytical reagent grade and were obtained from Sigma or Fluka (Buchs, Switzerland).

### aS dimers cloning and expression

The pET28b (Novagen) plasmid was used for the expression of recombinant human aS in *E. coli* BL21 (DE3). For the production of the dimers, the pET28b/aS plasmid was modified using standard cloning procedures. aS dimers are formed by two aS molecules, linked by their N-terminals (NN) or C-terminals (CC) segments. The formation of NN and CC dimers was obtained by the addition of a Cys residue at aS terminals and the formation of disulphide bonds. For NN, the Cys residue was introduced by V3C substitution, while for CC, GC residues were added at position 140 of aS. NC dimer, which links N- and C- terminals of aS, was produced as a single polypeptide molecule containing two aS sequences joined by RS residues. Similarly, DC dimer is a single polypeptide molecule, which contains 1–104 and 29–140 residues of aS. The expression and purification of all the recombinant proteins were conducted using the procedure previously described [28].

### Native-PAGE

Native polyacrilamide gel electrophoresis was performed on 12% polyacrylamide gel, with a constant current of 25 mA, using a Mini–PROTEIN II Bio-Rad electrophoresis system (Hercules, CA). 5 µg of protein were loaded into each well, and the protein bands were visualized by Coomassie blue staining.

### Chromatographic analyses

Reverse phase-high pressures liquid chromatography (RP-HPLC), was conducted on a Jupiter C4 column (4.6×150 mm, Phenomenex, USA). Elution was obtained with a linear gradient of acetonitrile (0.085% TFA) vs water (0.1% TFA), from 10 to 40% in 5 min and from 40 to 45% in 40 min at a flow rate of 1 ml/min. The identity of the protein material was assessed by mass spectrometry using an ESI-QTOF Micro instrument (Waters, Milford, MS, USA). Gel filtration chromatography (GF) was performed with a Superdex 200 10/300GL column (Amersham Biosciences, Uppsala, Sweden), using an ÄKTA FPLC system (Amersham Biosciences, Uppsala, Sweden), eluting at 0.4 ml/min in 20 mM in Tris-HCl, 150 mM NaCl, pH 7.4, and recording the absorbance at 214 nm. 50 µg of each protein samples (aS, NN, CC, NC and DC dimer), dissolved in the eluting buffer, were loaded into the column. The hydrodynamic volume of the analytes was determined on the basis of their distribution coefficients, Kd, calculated as the following formula: Kd = (V_e_-V_0_)/(V_t_-V_0_), where V_e_, V_t_ and V_0_ are the standard proteins, the total and the void elution volume, respectively. The calibration function (y = 1.15–0.38×) was obtained by plotting Kd against the logarithm of the molecular weight of proteins. A mixture of proteins of known MW was used (bovine α-lactalbumin, 14 kDa; carbonic anhydrase, 29 kDa; ovalbumin, 45 kDa; bovine serum albumin, 66 kDa; β-amylase, 200 kDa; apoferritin, 443 kDa and, thyroglobulin, 669 kDa). Blue dextran and 0.05% dimethyl sulfoxide (DMSO) were loaded to estimate V_0_ and V_t_.

### Circular Dichroism

Protein concentrations were determined by absorption measurements at 280 nm using a double-beam Lambda-20 spectrophotometer (Perkin Elmer, Norwalk, CT). The extinction coefficients of the proteins at 280 nm are 5960 (aS), 12045 (NN and CC dimer), 11920 (NC dimer) and 7450 M^−1^⋅cm^−1^ (DC dimer), as evaluated from their amino acid composition by the method of Gill and von Hippel [29]. Circular dichroism spectra were recorded on a J-710 spectropolarimeter (Jasco, Tokyo, Japan). Far-UV CD spectra were recorded using a 1 mm path-length quartz cell and a protein concentration of 3–10 µM. The mean residue ellipticity [θ] (deg⋅cm^2^⋅dmol^−1^) was calculated from the formula [θ] = (θ*_obs_*/10) ⋅(MRW/*lc*), where θ*_obs_* is the observed ellipticity in deg, MRW is the mean residue molecular weight of the protein, *l* the optical pathlength in cm and *c* the protein concentration in g/mL. The spectra were recorded in PBS (8 mM Na_2_HPO_4_, 137 mM NaCl, 2 mM KH_2_PO_4_, 2.7 mM KCl) buffer, pH 7.4. For SDS titration (ranging from 0–5 mM detergent concentration), protein concentration was 20 µM for aS, NN, CC and NC, and 6.7 µM for DC dimer.

### Fourier transformed infrared spectroscopy

Deuterated aS and dimers were prepared by three cycles of dissolution in D_2_O, filtration with a 20 nm pore-size filter (Whatman, Maidstone, UK), freezing of the protein solution at −80°C, followed by lyophilisation. The spectra of aS and dimers in solution were registered after dissolving the deuterated proteins in 20 mM Tris·DCl, 150 mM NaCl, pH* 7.2 (uncorrected for isotopic effects) at a concentration of ∼5 mg/ml. Spectra were recorded at 20–22°C using a Perkin Elmer 1720× spectrometer (Norwalk, CT, USA), purged with a continuous flow of N_2_ gas. Protein samples were placed between a pair of CaF_2_ windows separated by a 50 µm Mylar spacer. For each protein sample, 50 interferograms were accumulated at a spectral resolution of 2 cm^−1^. The spectra were analyzed using the Grams 32 program version 4.14 (Galactic Industries Corporation, Salem, NH). Buffer spectra were recorded under identical conditions to those used for protein samples and subtracted from the spectra of the latter. The second derivative of the amide I and II bands was used to identify the different spectral components. Thereafter, curve fitting was performed with Gaussian and Lorentzian line shapes, and with bandwidths varying between 15 and 25 cm^−1^ [30], [31].

FT-IR spectra of dimer ultra-centrifuged fibrils (90000 rpm for 2 hours at 4°C) were also recorded. Fibrils were obtained after 4–6 weeks of incubation of deuterated protein in the buffer previously described, at a protein concentration of 70 µM, at 37°C under shaking at 500 rpm in a thermo-mixer (Compact, Eppendorf, Hamburg, DE).

### Nuclear Magnetic Resonance

The expression of aS dimers for NMR studies (^15^N-labeled proteins) was achieved by growing cells in M9 minimal medium. Heteronuclear Single Quantum Correlation (HSQC) spectra were acquired on a Bruker Avance DMX spectrometer equipped with a gradient triple resonance probe. ^15^N-labelled aS samples (100–350 µM) were dissolved in PBS buffer containing 10% D_2_O. The experiments (256 increments of 512 time points each) were acquired at 283 K with 16 transients. The spectral widths were 3 ppm (^1^H) and 22 ppm (^15^N) and the frequency offsets were 8 ppm (^1^H) and 116 ppm (^15^N). Prior to Fourier transformation, the data were multiplied by a 90° shifted *sin* function in both dimensions. Spectra were processed using MestReC software.

### Fluorescence Polarization aggregation based kinetic

70 µM aSG141C142 was mixed with 5 molar excess of tris(2-carboxyethyl)phosphine in 20 mM Tris buffer, pH 7.0. After 30 min, 5 fold molar excess Oregon Green 488 maleimide (Molecular Probes, Invitrogen, CA, USA) previously dissolved in 20 mM Tris pH 7.0, was added to aSG141C142 solution. The reaction was held 4 hour at 45°C, then was analyzed by RP-HPLC by using a Jupiter C4 column (Phenomenex, CA, USA). Elution was obtained with a linear gradient of acetonitrile (0.085% TFA) vs water (0.1% TFA), from 39 to 46% in 14 min at a flow rate of 0.6 ml/min. Fluorescence polarization (FP) measurements were performed with a fluorescence plate reader DTX 880 Multimode Detector (Beckman Coulter, IN, USA), equipped with an excitation filter at 485 nm and two emission filters at 535 nm. Excitation and emission filters were equipped with polarizer. Measurements were carried out at 37°C, 1000 rpm shaking. 1 mg/ml of protein species was dissolved in PBS, 0.05% sodium azide, and mixed with 1/100 molar ratio of aSG141C142 labelled with Oregon Green 488. Time points were collected every 6 or 12 hours and FP (y) values were plotted against the incubation time (t). The data were fitted with a sigmoidal curve that allowed to evaluate the slope at the flex point (t50) and the length of the lag-phase.

### Aggregation studies

Protein aggregation was carried out in PBS buffer. The protein solutions were filtered with a 0.22 µm pore-size filter (Millipore, Bedford, MA, USA) and incubated at 37°C for up to 14 days at a protein concentration of 70 µM (1 mg/ml), under shaking at 500 rpm in a thermo-mixer (Compact, Eppendorf, Hamburg, DE). Aliquots of the samples were withdrawn during incubation for further analysis.

The ThT binding assays were performed accordingly to LeVine [32] using a freshly prepared 25 µM ThT solution in 25 mM sodium phosphate (NaH_2_PO_4_), pH 6.0, that had been passed through 0.22 µm filters. Aliquots (30 µl) of protein samples from the aggregation mixture were taken at distinct times and diluted into the ThT buffer (final volume 500 µl). Fluorescence emission measurements were performed at 25°C using an excitation wavelength of 440 nm and recording the ThT fluorescence emission spectra between 460 and 560 nm. Fluorescence intensities were plotted against the incubation times and fitted with a 4 parameter sigmoid curve (Sigmaplot software), using the following formula: y = y_0_·{a/[1+e^−(x-x0)/b^]}, and normalized by their plateau (a). The slopes were determined as a/4b.

### Transmission Electron Microscopy and Atomic Force Microscopy morphological analysis

Aliquots of the aggregation samples after 20 days of incubation were examined by transmission electron microscopy (TEM). The samples were diluted 3 times with PBS. A drop of the samples solution was placed on a Butvar-coated copper grid (400-square mesh) (TAAB-Laboratories Equipment Ltd, Berks, UK), dried and negatively stained with a drop of uranyl acetate solution (1%, w/v). TEM pictures were taken on a Tecnai G2 12 Twin instrument (FEI Company, Hillsboro, OR, USA), operating at an excitation voltage of 100 kV).

For AFM analysis, aliquots (10 µl) from aggregation mixtures were diluted in 90 µl of PBS buffer, left to equilibrate at room temperature for 10 min then deposited on freshly cleaved mica (RubyRed Mica Sheets, Electron Microscopy Sciences, Fort Washington, USA) and left to adsorb for 2 min. The mica surface was then rinsed with ∼500 µl of ultrapure water and dried under nitrogen. AFM imaging was performed in tapping mode with NSC15 phosphorous-doped silicon probes (MikroMasch, Tallin, Estonia) on a NanoScope IIIa SFM system equipped with a Multimode head and a type E piezoelectric scanner (Veeco, Santa Barbara, CA, USA). Raw SFM images were processed only for background removal (flattening) using Gwyddion v2.26. Diameters of the aggregates were measured from the images via a custom script [33] that automatically recognizes fibrils and interpolates their shape with a cubic B-spline function. The Z scale values of the individual pixels crossed by the B-spline in the AFM image were pooled to obtain the distributions of the apparent diameters.

### Proteolysis of fibrils

Proteolysis experiments were carried out on ultra-centrifuged (90000 rpm for 2 hours at 4°C) aggregation samples obtained after 1 month of incubation. Fibrils were dissolved in PBS, pH 7.4 and treated with proteinase K or porcine trypsin (E/S ratio of 1∶1000 and 1∶50, by weight, respectively). The reactions were quenched after 2 hours by acidification with TFA in water (4% v/v). The proteolysis mixtures were ultra-centrifuged. The soluble fractions were directly analyzed by RP-HPLC, while the pellets were incubated over night with 7.4 M Gnd-HCl before the analysis. RP-HPLC analyses were conducted using a Vydac C18 column (4.6 mm×250 mm; The Separations Group, Hesperia, CA), eluted with a gradient of acetonitrile/0.085% TFA vs. water/0.1% TFA from 5 to 25% in 5 min, from 25 to 28% in 13 min, from 28 to 39% in 3 min, from 39 to 45% in 21 min at a flow rate of 1 ml/min. The sites of cleavage along the polypeptide chains were identified by mass spectrometry analyses of the protein fragments purified by RP-HPLC.

## Results

### Chemical characterization

The produced aS dimers were identified by MS and isolated to homogeneity. Their chemical characterization is thoroughly described in the Supplementary Material (Fig. S1 and Table S1). The relevant information that emerged relates to the absence of aggregates in the purified material and to the absence of hints of acquired structure if comparing both the hydrodynamic radius and their electrophoretic mobility.

### Structural characterization of dimers in solution

**Figure 1:**
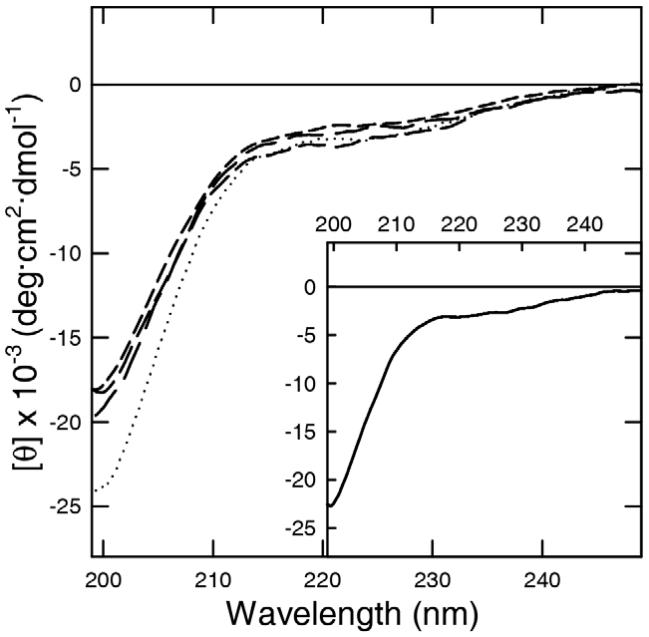
Far UV CD of aS dimers. The spectra were recorded in PBS buffer pH 7.4 at a protein concentration of 5 µM, using a quartz cuvette with 1 mm of pathlength. NN, CC, NC and DC are represented respectively by a long, medium, short dash and dotted line. Inset: Far UV CD of aS (continuous line).

The secondary structure of NN, CC, NC and DC in PBS buffer was evaluated by far-UV CD, FT-IR and NMR spectroscopy. The far UV CD spectra, recorded between 198 and 250 nm (Fig. 1) show a marked minimum at ∼200 nm corresponding to random coil structure, in analogy to aS [34].

**Figure 2:**
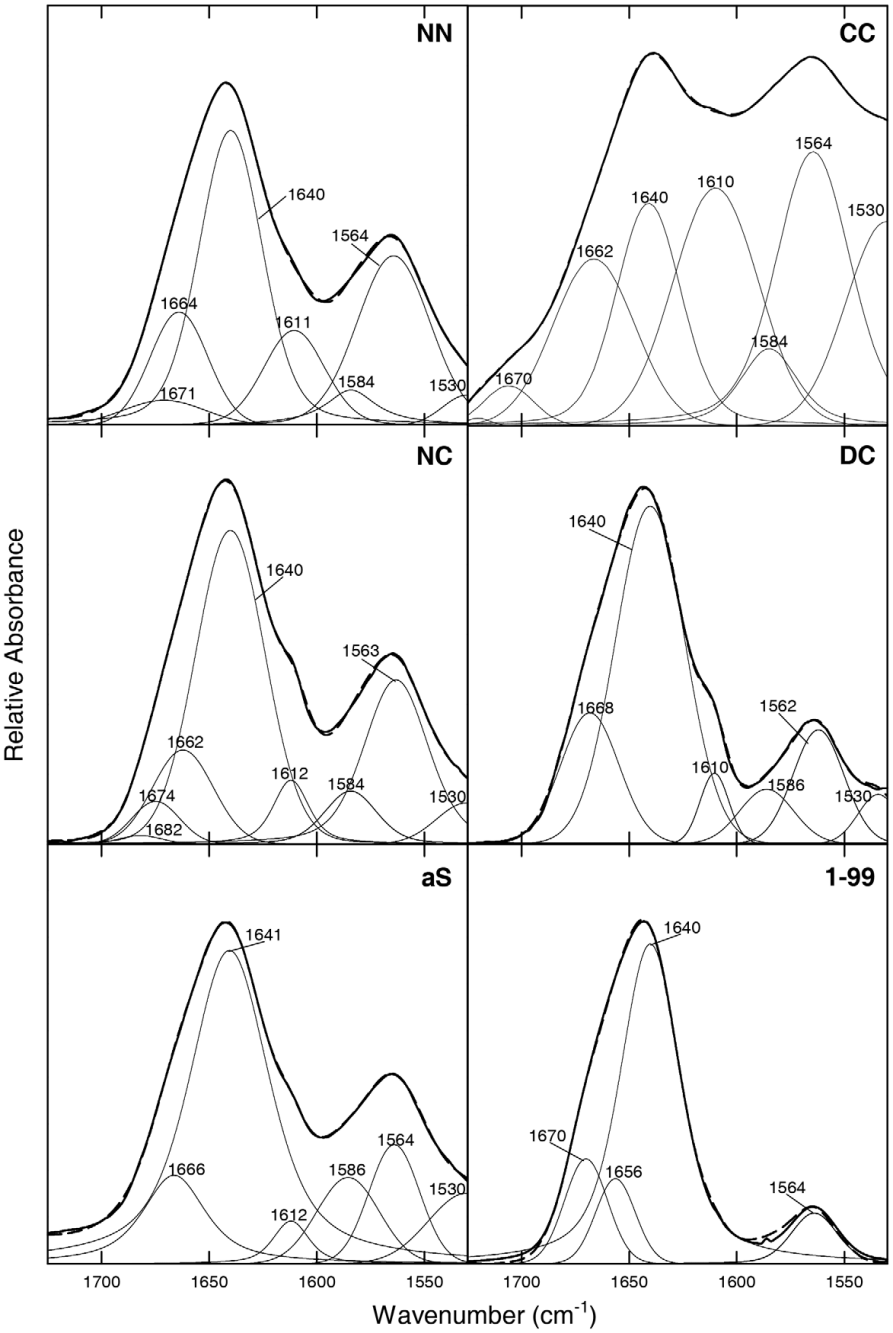
FT-IR spectra of aS dimers at pH* 7.2. Curve fitting was performed with Gaussian and Lorentzian lineshapes and with bandwidths varying between 15 and 20 cm^−1^. The peak position of the amide band components was deduced from the second derivative spectra (Fig. S2). The sum of the fitted curves is shown as a broken line, closely overlapping the experimental trace, shown as a continuous line.

For FT-IR spectra, deuterated proteins were dissolved in a saline buffer (20 mM Tris·DCl, 150 mM NaCl, pH* 7.2, uncorrected for isotopic effects), and the IR absorbance was recorded between 1500 and 1750 cm^−1^ to evaluate the contribution of both Amide I and II bands (Fig. 2, bold lines). The second derivatives of the curves allowed identifying the different spectral components (Fig. S2), which were used for the curve fitting procedure (Fig. 2, thin lines and Table 1). Table 1 reports the percent contributions of the different types of secondary structure, calculated as the ratios between the areas of the curves corresponding to the distinct component bands and the total area underneath the spectrum. The main band of NN, NC and DC FTIR spectra in the Amide I region is at 1640 cm^−1^ (38–54% of the total area, Table 1), which corresponds to the vibrational motions of the backbone amide moieties in random conformation. The same band is observed for aS, in agreement with previous results [12]. At variance, the intensity of this band in CC spectrum is lower (17%). Another secondary structure component in all dimers is indicated by the presence of a band at 1656–1671 cm^−1^ associated to turns, which accounts for 12–17% (Table 1). The bands of deprotonated carboxylic moieties of Glu (1560–1568 cm^−1^) and Asp (1580–1586 cm^−1^) residues in the Amide II region are also intense (Table 1). The Amide II band is sensitive to secondary structure but also to the hydration state and to the hydrogen bonding of the protein side chains [35]–[37]. In the IR spectrum of CC dimer the major contribution derives from the band at 1530 cm^−1^ (17%), that specifically corresponds to β-sheet or β-turn structure [38]–[40] and indicates amide protons in strongly hydrogen bonded structures [41]. To better define the contribution of Glu and Asp side chains (bands at 1568–1586 cm^−1^), the FTIR spectrum of the C-terminus truncated form of aS (sequence 1–99) was recorded (Fig. 2, Table 1). The main band is at 1640 cm^−1^ in the Amide I region (69%) due to random conformation, along with two smaller bands indicative of turns (1656 and 1670 cm^−1^, 23%). Of interest, the removal of the C-terminal region, where the highest number of Glu and Asp carboxylic moieties is present, causes a significant decrease in the intensity of the Amide II band (8%, if compared to 21–31% of aS and its dimers).

**Table 1:** Secondary structure content of aS dimers as determined by FTIR spectroscopy analysis reported in Fig. 2.

*Wavenumber* a *(cm^−1^)*	*Structural Assignment*	*aS %* b	*NN %* b	*CC %* b	*NC %* b	*DC %* b	*1–99 %* b
1530	β-sheet/turns	10	3	17	5	3	-
1560–1568	Glu (COO^−^)	12	25	25	22	14	8
1580–1586	Asp (COO^−^)	9	5	6	7	7	-
1610–1612	Tyr/β-sheet	-	12	21	12	5	-
1640	Random	55	38	17	42	54	69
1656–1671	Turns	14	17	14	12	17	23

a Peak position of the amide I band components, as deduced by the second derivative spectra.

b Percentage area of the amide I band components, as obtained by integrating the area under each deconvoluted band.

**Figure 3:**
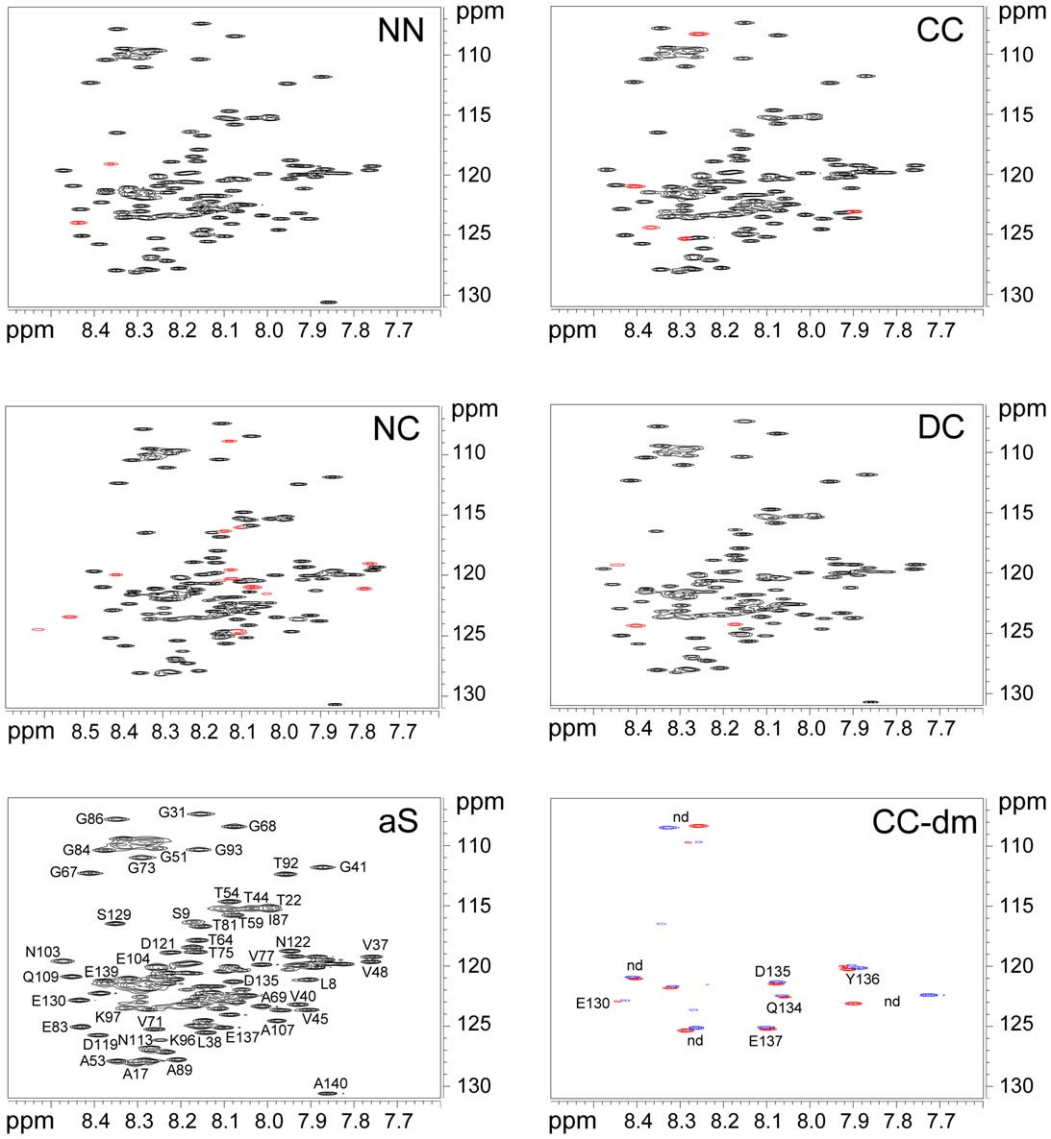
HSQC spectra of NN, CC, NC and DC dimers. aS HSQS spectrum is reported with resonance assignments, where space permits. In red are indicated new peaks of dimers spectra that are not present in aS spectrum. For CC dimer, experiments were performed in the absence and in the presence of 10 mM DTT and the difference maps (CC-dm) was calculated by subtracting the data matrix obtained after 2 hours of incubation in the presence of DTT from the reference.

To verify whether dimerization can promote interactions, ^1^H-^15^N HSQC spectra of aS dimers were recorded (Fig. 3). All the spectra exhibit a dense cluster of cross-peaks over a narrow range, corresponding to largely unfolded proteins and suggesting that each dimer presents the same behavior of aS in solution. Spectra of NN, CC and DC dimers are very similar to the spectrum of aS with the appearance (and disappearance) of a very small number of peaks, corresponding to the amino acids of the terminals involved in the linkage of the dimer. In agreement with the fact that the chemical environment of both the C-terminal tail of one monomer and the N-terminus of the other one are modified in the dimer linkage, the spectrum of the NC dimer shows a larger number of differences with respect to that of aS. In order to assess if small not easily detectable differences are present in the CC dimer, in which FTIR spectra indicate a contribution of β-sheet and absorption bands assigned to side chains, DTT was added inside the NMR tube and an HSQC spectrum of the reduced CC was recorded. The difference map, calculated by subtracting the data matrix obtained after incubation in the presence of DTT from the reference one, indicates that the effects are related to the C-terminal portion and do not involve other regions of the protein.

### Interaction of dimers with SDS

**Figure 4:**
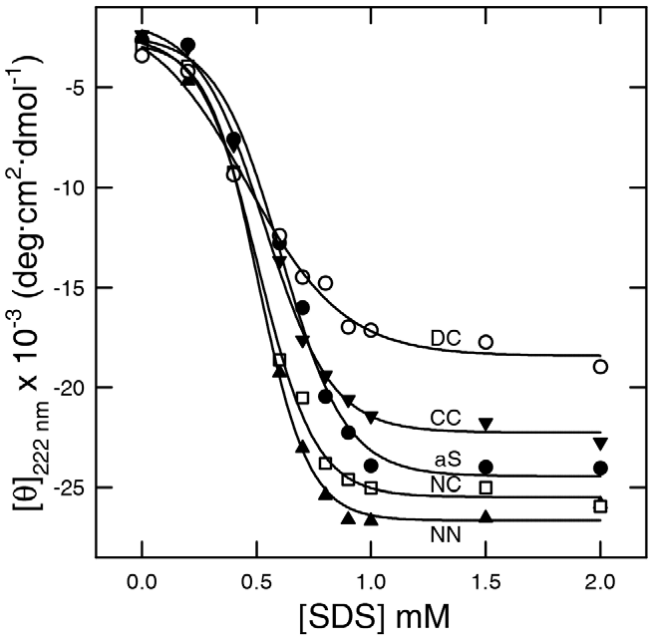
Ellipticity at 222 nm ([θ]_222 nm_) as a function of SDS concentration (mM) deduced from the spectra reported in Fig. S3. NN, CC, NC and DC are represented respectively by black triangle up, black triangle down, empty square, empty circle, respectively. SDS dependence curve of aS is also reported (black circle).

aS is able to acquire a α-helix conformation in the presence of SDS. In the titration experiments over a range of SDS concentration (0–2 mM), the presence of an isodichroic point at 203 nm suggested for aS a simple two-state conformational transition between random coil and α-helix [42]. The effect of SDS on the secondary structure of aS dimers was evaluated by far-UV CD spectroscopy (Fig. S3), showing that all dimeric species described here acquire α-helix structure in a simple two-state conformational transition. The protein ellipticity at 222 nm was plotted as a function of SDS concentration and it follows for all dimers a sigmoidal trend (Fig. 4, Table S2), suggesting a strict dependence of the structural transitions on SDS concentration. Initial addition of SDS (0.2 mM) results in minimal changes in protein secondary structure. Increases in SDS concentration from 0.2 to ∼1 mM induce the acquisition of α-helical structure. Further addition of SDS (1–5 mM) does not perturb the protein secondary structure. Within the concentration range analyzed here (up to 2 mM SDS) the final amount of α-helical structure is slightly larger for NN and NC than CC (NN∼NC>aS>CC) (see Table S2, y_0_ value). Moreover, a scale of propensity of the structural transition was established and it is in the order NN>NC>aS>CC (see Table S2, slope). DC dimer shows a moderately different behavior. The ellipticity at 222 nm of this dimer is ∼−17.000 deg·cm^2^·dmol^−1^ at 1 mM SDS concentration.

### Aggregation process of aS dimers

**Figure 5:**
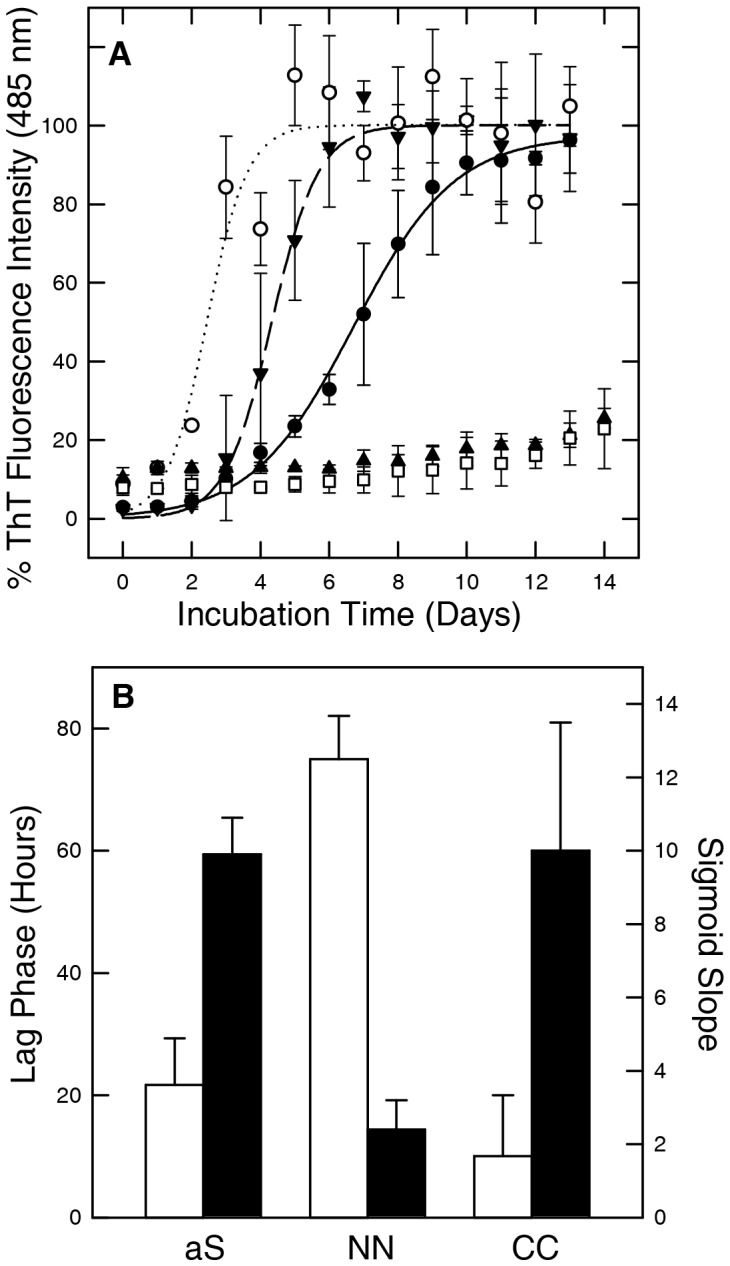
Time-course analysis of the aggregation process of aS dimers, followed by ThT fluorescence assay (A) and fluorescence polarization (B). The aggregation processes were conducted at a protein concentration of 1 mg/ml. Fluorescence intensity of ThT is reported as percentage of the plateau of emission intensity corresponding to each curve. NN, CC, NC and DC are represented respectively by black triangle up, black triangle down, empty square, empty circle, respectively. aS aggregation curve is indicated by black circles. Error bars were calculated from three independent aggregation experiments. For FP, lag-phase, (white bars) and curve slope at t50 (black bars) for NN, CC and aS were reported. The values of lag phase and slope were deduced from four independent aggregation experiments.

The kinetics of fibril formation of aS dimers was monitored using Thioflavin T (ThT) fluorescence (Fig. 5). All dimers are able to form amyloid-like aggregates and show an increase of ThT fluorescence at 485 nm. Aggregation of DC (empty circles) and CC (black triangles down) follows a sigmoidal-shaped ThT fluorescence curve similar to that of aS (Fig. 5A, black circles). DC and CC show a lag phase of less than 24 and 48 hours, respectively followed by a rapid growth phase that reaches a plateau at 4 and 6 days, respectively. aS has a lag phase of 48 hours and a plateau is reached after 10 days. A difference between DC, CC and aS kinetic properties lies in the slopes of the fitted sigmoid curves which are 51.2±6.4, 41.5±5.8 and 18.1±7.2, calculated at the flex position. NN and NC dimers show quite slow aggregation kinetics, and in the considered time range (15 days), no significant increase of ThT fluorescence is observed. ThT fluorescence starts to increase after 4 weeks (not shown).

The aggregation properties of NN and CC dimers were tested with a complementary technique based on fluorescence polarization (FP), which intensity is proportional to the diffusion coefficient of the fluorescent probe linked to the protein [43]. However, in this case the increased FP signal only witnessed the increase in size of oligomers rather than fibrils, being the fluorescent probe equally immobilized in large oligomers and fibrils [43]. For both dimers, in analogy with aS, an increase of FP over time with a sigmoidal trend was observed (not shown). In Fig. 5B we report the lag phase preceding FP increase and the curve slope at t50. The first parameter is related to the time required to the early aggregation events to generate detectable oligomeric nuclei. The curve slope is linked to the growing rate of oligomers. CC has a aggregation kinetic similar to aS. NN presents a three folds increased lag phase (Fig. 5B, white bar), indicating a longer time required for the formation of detectable oligomeric nuclei than aS and CC. Moreover, its slope is less steep than the ones observed for aS and CC, suggesting that, once nuclei are formed, monomer addition to growing oligomers is less favored in comparison with aS (Fig. 5B, black bar).

### Structural and morphological characterization of fibrils

**Figure 6:**
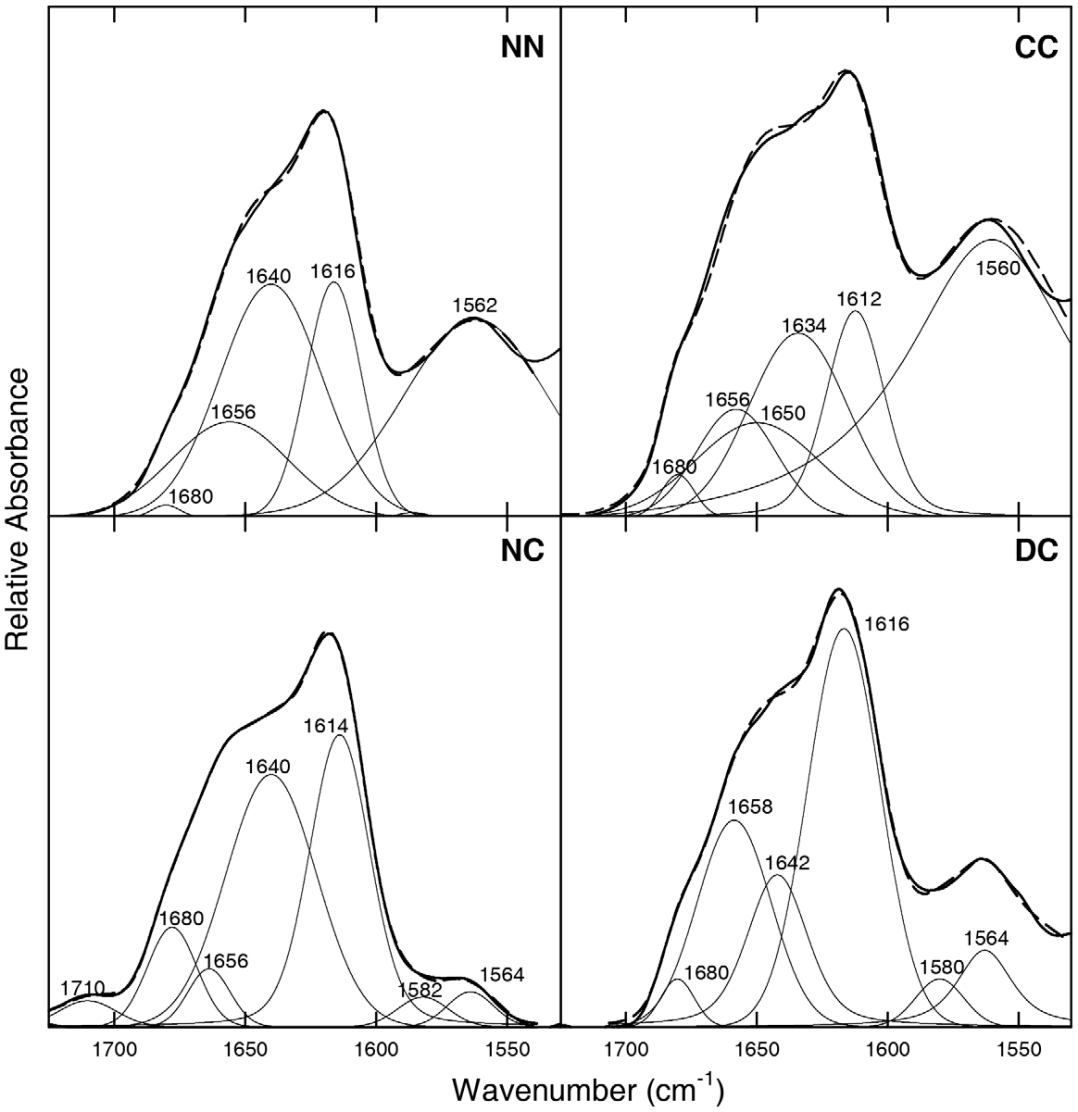
FT-IR spectra of aS dimer fibrils. Protein fibrils obtained after one month of incubation were dissolved in 20 mM Tris·DCl, 150 mM NaCl pH* 7.2. The peak position of the amide band components was deduced from the second derivative spectra (Fig. S4). Curve fitting was performed with Gaussian and Lorentzian lineshapes. The sum of the fitted curves is shown as a thin dashed line, closely overlapping the experimental trace, shown as a continuous bold line.

FT-IR spectra were recorded to evaluate the type and content of secondary structure in the proteins aggregates (Fig. 6, Table 2). All samples were first deuterated and then aggregation was induced for an incubation time of 4 weeks. Afterwards, samples were ultra-centrifuged, to remove the contribution of non-aggregated species and obtain, upon resuspension of the fibrilar material, optimal concentrations for the measurement. The spectra of the aggregated dimers and of aS show the characteristic band at 1612–1616 cm^−1^, which corresponds to the vibrational motions of the backbone amide moieties in aggregated cross β-sheet structure, and accounts for 13–35% of the total area. A further contribution of secondary structure in all the fibrils is due to the presence of turns (1659–1668 cm^−1^ band, 9–22%) and unordered regions (1640–1650 cm^−1^ band, 11–38%). The latter band (unordered structure) was observed also with other protein fibrils [44]. The CC dimer shows a large contribution deriving from Glu side chains (48%), suggested by the presence of the 1560–1568 cm^−1^ band, whereas in the same dimer before aggregation this contribution accounted for 25%. In NC an opposite behavior is observed, since the contribution of Glu and Asp side chains almost disappears following aggregation (6% in total compared to 29% before aggregation).

**Table 2:** Secondary structure content of aS dimers fibrils as determined by FTIR spectroscopy reported in Fig. 6 and in Fig. S5.

*Wavenumber* a *(cm^−1^)*	*Structural Assignment*	*aS %* b	*NN %* b	*CC %* b	*NC %* b	*DC %* b
1560–1568	Glu (COO^−^)	23	33	48	3	8
1580–1586	Asp (COO^−^)	5	-	-	3	4
1612–1619	Aggregated β-sheet	31	18	13	32	46
1634–1636	β-sheet	18	-	18	-	-
1640–1650	Random	-	33	11	38	16
1656–1668	Turns	19	15	9	22	24
1680–1689	Anti-parallel aggregated β-sheet	4	1	1	2	2

a Peak position of the amide I band components, as deduced by the second derivative spectra.

b Percentage area of the amide I band components, as obtained by integrating the area under each deconvoluted band. The areas corresponding to side chain contributions located at 1700–1710 cm^−1^ have not been considered.

**Figure 7:**
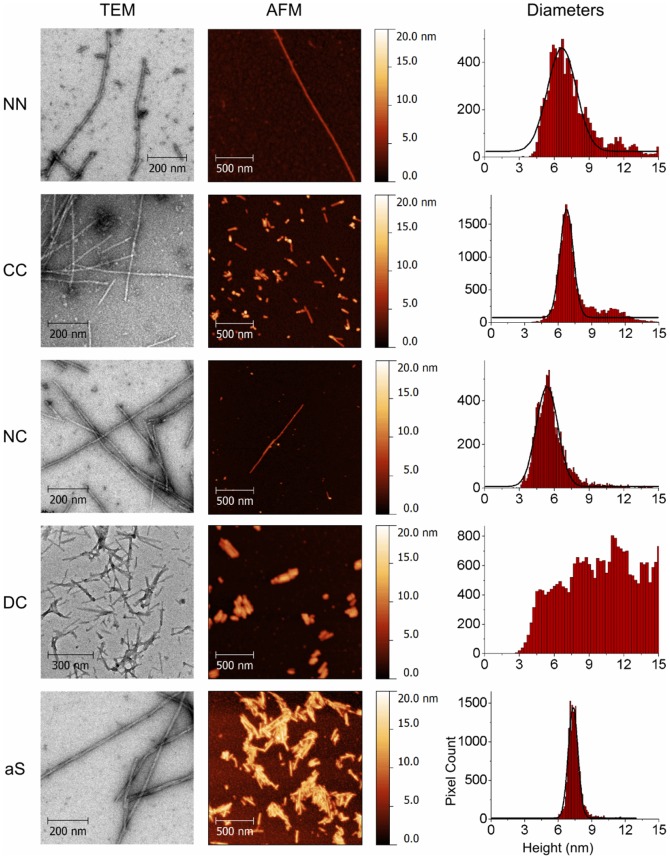
Representative TEM and AFM images of fibrils formed by the NN, CC, NC and DC dimers (rows 1–4) and aS (fifth row) (left and central columns). Right column: distributions of the apparent diameters of the fibrillar aggregates, as measured from AFM images via an automated procedure (see main text). Gaussian fits are shown (solid black lines) where applicable.

The morphology of aggregates formed by all the dimers was investigated by transmission electron (TEM) and atomic force (AFM) microscopy (Fig. 7), indicating that all the aggregates appear as amyloid-like fibrils. Fibrillar aggregates formed by NN, CC and NC are long and straight, showing a range of lengths spanning from tens of nanometers to microns, and have relatively constant diameters along the fibril axis. Conversely, fibrils obtained from DC are shorter, reaching a maximum length of approximately 0.5 microns, and show a more irregular appearance along their main axis. The distributions of the aS and NC fibril diameters as estimated by AFM imaging are well fitted by single Gaussian functions peaked at 7.3±0.8 nm and 5.4±0.9 nm, respectively. aS value is in accord with the results of similar experiments performed in air [45]. Although NN and CC show broader distributions than aS and NC, single Gaussian fits peaked at 6.7±1.4 nm (NN) and 6.7±0.7 nm (CC) still adequately capture the vast majority of the observed diameter values. DC fibrillar aggregates show instead a broad, almost random distribution of different diameters (Figure 7, right column). TEM images show larger diameters, accordingly with literature data [46], and are about 9.4±1.3 nm for a single filament. For DC fibrils, TEM shows a diameter of 11.8±1.3 nm, suggesting a larger section.

Fibrils formed by aS have a measurable periodicity of 82±7 nm as estimated by AFM height profiles, and clearly appear as formed by two left-hand-twisted filaments in TEM images. Fibrils formed by NN and CC dimers instead appear as single filaments even at the maximum AFM and TEM resolutions available to us. NC fibrils rarely show a periodicity of ∼80 nm, suggesting that they are also formed by twisted sub-fibrillar filaments, but the vast majority of NC aggregates does not show such features, thus hindering statistically significant morphological observations on this specific aspect (Fig. S6). A striking correspondence between the diameter of NC fibrils and the individual filaments, often called protofibrils, composing aS mature fibrils is evident. While the vast majority of aS aggregates is constituted by mature fibrils having a diameter of around 7.3 nm, aS protofibrils, either sprouting from a mature fibril or isolated are rarely observed (Fig. S7). The measured diameter of the aS protofibrils (5.4±0.6 nm) closely matches the diameter of NC fibrils (5.4±0.9 nm), suggesting that both aggregates are structurally similar, although aS protofibril is extremely prone, via lateral adhesion, to further aggregation into larger fibrils.

### Identification of the core structure of dimers fibrils by proteolysis

**Figure 8:**
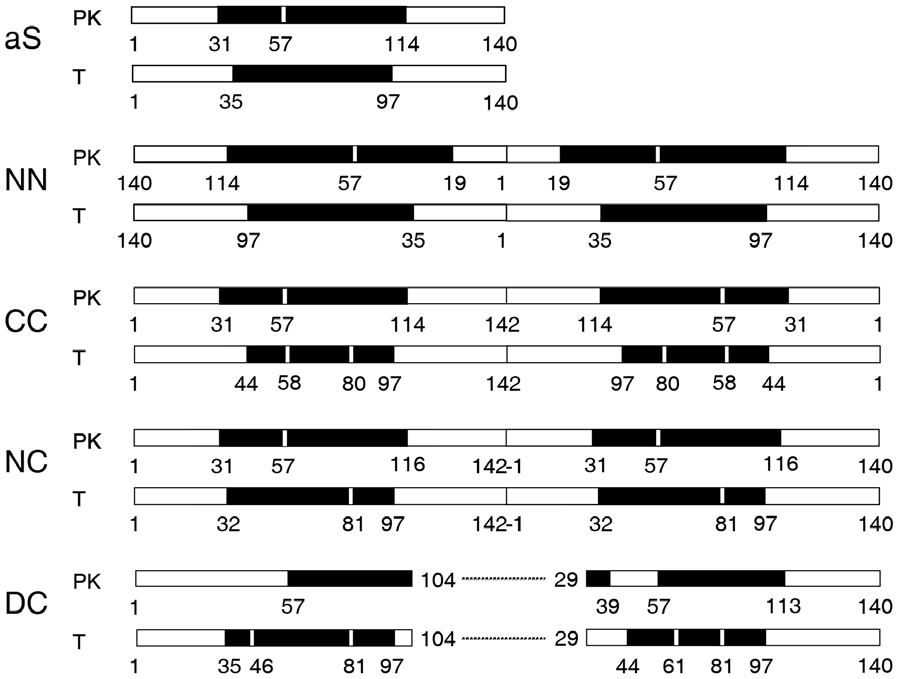
Scheme of proteolytic sites of aS dimer fibrils by using proteinase K (PK) and trypsin (T). Sequences cleaved off are represented in white, protected regions are indicated in black. The numbers refer to N-terminal of each peptide. As DC is constituted by the sequence 1–104 linked to 29–140 of aS, the sequence notation of its proteolytic fragments refers to the aS sequence numbering. In the case of fragments encompassing the two sequences, these were indicated with numbering of the segments deriving from both sequences separated by a slash.

To define the region(s) of the proteins involved in the core of the fibrils formed by the different types of dimers, an approach based on proteolytic digestion coupled to mass spectrometry was used. Regions of the protein sequence normally exposed to proteases exhibit limited accessibility when involved in β-sheet structure [44], [47], [48]. Proteinase K (PK), which displays broad substrate specificity [49], and trypsin (T), which specifically hydrolyzes peptide bonds containing basic residues at the C-terminus, were used. The digested fibrils were checked by TEM and they show morphologies quite similar to those of the undigested fibrils (not shown). The peptides obtained after digestion were ultra-centrifuged, and pellet and supernatant analyzed separately by RP-HPLC. Peptides were identified by ESI mass spectrometry (Tables S3 and S4). As a control experiment, proteolysis was conducted also on freshly dissolved monomeric dimers species and aS under the same proteolysis conditions used for the digestion showing an almost complete degradation (not shown). The analyses of the proteolytic patterns of the fibrils obtained both from aS and the different full length dimers did not evidence remarkable differences in the proteolytic patterns, as shown in Fig. 8. The pattern of aS fibrils digested by PK evidences a region resistant to proteolysis that encompasses amino acids from 31 to 113, with a cleavage site between residues 56–57 in agreement with previous results [50]–[51]. A similar region was isolated after trypsin digestion (region from 35 to 96). For NN, CC and NC dimer the region resistant to proteolysis is almost the same (Table S3, S4). Notably, there are not peptides belonging to the protected region in the soluble fractions after ultracentrifugation, suggesting that both molecules that constitute the dimer are involved into the core structure.

DC dimer sequence is constituted by 1–104 linked to 29–140 of aS. The sequence notation reported for its proteolytic fragments refers to aS sequence numbering, therefore, the fragments encompassing the two sequences were indicated with the numbering of the segments deriving from both sequences separated by a slash. Results obtained from both PK and trypsin digestions show that the connecting loop between the two NAC segments (only 15 residues) is susceptible to proteases attack (Fig. 8). As a consequence, it is not involved in the stable secondary structure, as in the cross-β-structure of fibrils core. Moreover, some hydrolysis sites were also detected in the region expected to be protected (60–61 and 80–81).

## Discussion

This study was aimed to address two issues. The first one refers to the possibility to speed up the aggregation process thought an increase of local concentration of aS by generating covalent dimers. The second issue explored is the topology of the individual aS molecules as building blocks of the aggregated fibers. aS dimers formation has been reported in the literature as product of redox chemistry, as described as an example for Tyr125 based dimerization upon nitrative stress [26]. The formation of dimers was also reported *in vitro*, cellular and animal models for Y39C and Y125C aS mutants. In this case the cysteine substitutions at the reported positions in the aS sequence were proposed to increase dimers formation and accelerate protein aggregation and cellular toxicity [52], [53] probably through the formation of oligomeric species.

In our study the procedure used to produce aS dimers relies on two strategies: the first was focused on the formation of dimers with a minimal number of constraints on the fibrillation process. aS molecules were linked with different reciprocal orientations through their N-terminals (V3C), the C-terminals (adding 141G and 142C) or directly cloned in tandem. The second strategy led to the production of a fourth dimer, in which the two fibrils core regions (residues 1–104/29–140) were juxtaposed. The dimerization of aS *per se* is neither sufficient to trigger the conversion to beta structure of the individual aS molecules nor to trap a transient potential beta formed in one of the two molecules of the dimers. NMR analysis of the structures of all produced dimers did not show any relevant difference with aS. The only modifications in the NMR spectra could be referred to the covalent link between the two monomers. Analogous results were obtained in the CD and IR experiments. Moreover, the intrinsic propensity of unfolded aS to acquire alpha-helix conformation in the presence of membrane-mimetic systems, such as SDS, is maintained in the dimer molecules.

The kinetic properties of the aggregation process, for the different dimer types, were first analyzed in terms of fibril formation. The ThT assay, that reports the growth of fibrils, showed only for the DC an aggregation behavior characterized by the presence of a shorter lag phase in comparison to length observed for aS and all other dimers, thus suggesting that the all the latter aggregate following a qualitatively similar nucleated polymerization process. Differences emerged in the fibril elongation phase starting immediately after the lag phase, as the CC dimer presented a steeper rise in the ThT fluorescence signal, which could be interpreted as a facilitated accretion of the dimer building blocks on the fibrils as a consequence of the topology of the individual aS molecules in the fibrils. This possibility will be discussed again later within the context of the proposed structural model for the fibrillar assemblies. The DC dimer presents a more aggregation prone behavior, but the end product seems to differ from the mature fibrils formed by aS, an observation that hinders the direct comparison of the kinetic data from these species.

To explore an earlier stage in the aggregation process, we analyzed the formation of oligomers as detected by fluorescence polarization assay. Using a protocol described by Luk et al. [43], we first monitored the formation of aS oligomeric species by following the changes in the value of fluorescence polarization of reporter labelled monomeric aS (1% of the dimers). The formation of oligomeric species was not accelerated by dimerization, the CC dimers presented the same lag phase and slope of the raising part of the fluorescence polarization intensity curve of the wt aS. Furthermore, the NN dimer, that in the ThT assay showed a slow rate of increase in fluorescence in comparison to aS, showed a significant increase in the length of the lag phase of oligomers formation. Taken together, these kinetic data suggest that the constraints posed by formation of covalent aS dimers act differently at the different stages of the aggregation process.

The quantitative morphological characterization of aS fibrillar products based on TEM and AFM images is in accord with the fibril structure model first proposed by Fink and coworkers [51], in which mature aS fibrils are formed by the entwining of two sub-components called protofibrils into a left-handed-twisted bundle. The striking coincidence between the diameters of aS protofibrils and NC fibrils (see Results section and Figure S7) allows an interesting observation on the aggregation process of aS mature fibrils. The fact that aS protofibrils are rarely observed, and are invariably either (i) branching out from mature fibrils, or (ii) very short if observed in isolation, suggest that during the aggregation of aS, the joining of protofibrils happens concurrently with their formation (and possibly elongation), and not afterwards as proposed in the so-called “hierarchical” aggregation models. In contrast, NC fibrils, although morphologically very similar to aS protofibrils, reach often lengths of microns, suggesting that their ability to side-joining is severely hindered with respect to their aS counterparts.

The structural characterization of the fibrillation products of the dimers may provide the rationale to contextualize the results presented here in the light of the recent SSNMR studies on aS fibrils. Several laboratories focused on the definition of the aS monomer structure within fibrils [54], [55]. The converging picture, with some inconsistency on the boundaries of the individual β-strands, identifies 5 consecutive strands going from residues ∼36 to ∼95. In this frame Vilar et al. [55], by evaluating the distance restraints between residues extracted from the ^13^C-^13^C proton driven spin diffusion spectra, proposed a relative topology for the individual strands that defines a fold for the monomer in which the five strands define a plane. This model of the fold of the monomer within the fibril has been recently confirmed by Pulsed EPR on a spin labeled aS, in which the distance between the residues 41/42 and 90/91 was estimated to range from 3,9 to 5,1 nm, according to relative orientation in the strands [56]. The stacking along the main axis of the fibers was deduced from the diffraction patterns described to be around 1,1 nm [57]. Starting from the structure deduced from the results described above, the constraints posed by the covalent dimerization in the different aS constructs to the building up of the fibers become informative. The NN and the CC present a long linker between the core regions of the two monomers of the dimers. The average diameters of their fibrils are very similar to aS and also the distribution of the diameter values is close to that of aS. In this case the linker connects the ends on the same side of the plane defined by the individual folded monomers. A relevant feature that is lost in both NN and CC dimers in comparison to aS is the diameter periodicity, previously ascribed to protofibrils twisting, as shown in Figure S6. In the case of the NC dimer the linking region, residues 96–140 of the first molecule and 1–36 of the following molecule, has to connect two points at the opposite side of the plane. This is not an issue in the structure of the core region, the IR is almost indistinguishable from that of aS, but the connecting loop hinders the formation of the twisted fibrils. This is documented by the shift to lower values of the heights distribution detected for the NC dimers. This observation hints on a role of either the N or the C term in the formation of the twisted structure, however, the absence of the twisted mature fibers and the substantial decrease in height observed for the C-terminal deletion mutant [58] strongly support the possibility that the latter is the determining feature.

The last evidence that provides further support to the model proposed by Vilar et al. [55], comes from the DC dimer. Again the IR of the DC based fibrils does not show any difference with aS suggesting that the core region secondary structure is conserved. However, the absence of a linker region between the end of the first folded monomer and the beginning of the sequence that should form the adjacent plane compromise the possibility to form the same type of fibrils that are formed by aS. Here, the engagement of one part of the dimer in the formation of a folded monomer in the fibril core leaves the second part of the DC molecule in wrong orientation to pile up along a growth axis of the fibril, the consequence is that the second part of the DC monomer may either be free or get involved in the formation of other parallel fibrils. This view is substantiated by the imaging data, which present a non-homogeneous distribution of heights for the DC base fibrils due to the superposition of growing fibrils.

In conclusion, this study allows to propose both a tentative scheme of the tertiary structure organization of the monomers in the fibrils, in which as previously proposed [55] they define a stacking of planar elements and to pinpoint a role for the non core region in the quaternary structure of the mature fibrils.

## Supporting Information

Table S1 **Chemical properties of aS dimers.**
(PDF)

Table S2 **Parameters gathered from the equation for the curves interpolating the dependence of the structural transition of aS dimers as a function of SDS concentration, as monitored by far-UV CD (** **Fig. 4** **).**
(PDF)

Table S3 **Analytical characterization of aS dimers fragments obtained by proteolysis with proteinase K of aggregated species, after removal of the soluble species by ultracentrifugation.**
(PDF)

Table S4 **Analytical characterization of aS dimers fragments obtained by proteolysis with trypsin of aggregated species, after removal of the soluble species by ultracentrifugation.**
(PDF)

Figure S1 **Chemical characterization of aS dimers by RP-HPLC (A), gel filtration (GF) chromatography (B) and native gel electrophoresis (C).** RP-HPLC was conducted on C4 column using a liner gradient of acetonitrile. GF experiments were conducted with a Superdex 200 column, eluted with Tris-HCl buffer (20 mM Tris, 150 mM NaCl, pH 7.4) at flow rate of 0.4 ml/min. aS, NN, CC, NC and DC are represented respectively by a continuous, long dash, medium dash, short dash and dotted line. GF calibration (D) was obtained using α-lactalbumin, carbonic anhydrase, ovalbumin, bovine serum albumin (BSA), β-amylase, apoferritin and thyroglobulin, as protein reference molecular markers (black dots). Proteins hydrodynamic volumes were calculated on the basis of their Kd (white dots). In the native PAGE, BSA was used as marker (first lane); NN_R_ and CC_R_ represent disulfide-bond containing dimers after reduction by β-mercaptoethanol.
(PDF)

Figure S2 **Second derivative of FT-IR spectra of aS dimers and 1–99 of the spectra reported in** **Fig. 2** **.** The relative values of the bands were used as reference for the curve fitting operation.
(PDF)

Figure S3 **Far UV CD spectra of aS dimers (A) and aS (B) in the presence of increasing concentration of SDS.** The spectra were recorded in PBS buffer pH 7.4 at a protein concentration of 20 µM. The numbers close to the curves indicate SDS concentrations (mM).
(PDF)

Figure S4 **Second derivative of FT-IR spectra of aS dimers aggregates reported in** **Fig. 6** **.**
(PDF)

Figure S5 **FT-IR spectra of aS fibrils obtained after one month of protein incubation.** The peak positions of the amide band components were deduced from the second derivative spectra (lower panel). The sum of the fitted curves is shown as a broken line, closely overlapping the experimental trace, shown as a continuous line.
(PDF)

Figure S6 **AFM images (left column) and height profiles along horizontal sections (right column) of representative individual fibrillar aggregates composed by each of aS dimers used in this study.** aS and NC height profiles are superimposed with a sinusoidal fit (solid red line). The best fitting sinusoid periods are 84 nm and 79 nm for aS and NC respectively.
(PDF)

Figure S7 **(A) AFM image showing two different types of aS fibrillar aggregates.** A rarely observed protofibril (*) lies near a mature fibril (**). (B) AFM image of representative NC dimer aggregates. (A1) Height profile of the fibrillar aggregates shown in panel A, measured along a broken line section (pale blue solid line in panel A). (A2, A3, B1) Distribution of all the measured diameters of mature aS fibrils (A2, **) aS protofibrils (A3, *), and NC fibrils (B1). Gauss fits are superimposed to each distribution (solid black lines) and are centered respectively on 7.3 nm (aS mature fibrils), 5.4 nm (aS protofibrils) and 5.4 nm (NC fibrils).
(PDF)
