# Supplementary material for: Covalent α-Synuclein Dimers: Chemico-Physical and Aggregation Properties
Source: PLoS One. 2012 Dec 13;7(12):e50027. doi: 10.1371/journal.pone.0050027 (PMC3521728; doi:10.1371/journal.pone.0050027)
Supplement: Table S1 — Chemical properties of aS dimers. (PDF) [file pone.0050027.s001.pdf]

Table S1. Chemical properties of aS dimers.

	amino acids	*Theoretical pI*	*Experimental MW (Da)*[a]	*Average MW (Da)*[b]
aS	140	4.7	14461.1 ±0.7	14460.1
NN	280	4.7	28926.3 ±0.5	28927.2
CC	284	4.7	29240.2 ±0.3	29239.6
NC	282	4.7	29145.5 ±0.1	29145.5
DC	216	5.2	21969.5 ±0.1	21969.7

[a] Experimental molecular masses determined by ESI-MS.

[b] Molecular masses calculated from the amino acid sequence of proteins.
