# Supplementary material for: Covalent α-Synuclein Dimers: Chemico-Physical and Aggregation Properties
Source: PLoS One. 2012 Dec 13;7(12):e50027. doi: 10.1371/journal.pone.0050027 (PMC3521728; doi:10.1371/journal.pone.0050027)
Supplement: Table S2 — Parameters gathered from the equation for the curves interpolating the dependence of the structural transition of aS dimers as a function of SDS concentration, as monitored by far-UV CD ( Fig. 4 ). (PDF) [file pone.0050027.s002.pdf]

Table S2. Parameters gathered from the equation for the curves interpolating the dependence of the structural transition of aS dimers as a function of SDS concentration, as monitored by far-UV CD (Fig. 4).

Protein	x_0[a]	y_0[b]	slope[c]
NN	0.51	-26600	-54400
CC	0.54	-22300	-35500
NC	0.51	-25500	-46300
DC	0.46	-18400	-20300
aS	0.61	-24400	-39100

[a] SDS concentration where 50% of protein population have acquired α-helical structure
[b] assumed ellipticity at infinite SDS concentration
[c] slope of the curves shown in Fig. 4
