# Supplementary material for: Covalent α-Synuclein Dimers: Chemico-Physical and Aggregation Properties
Source: PLoS One. 2012 Dec 13;7(12):e50027. doi: 10.1371/journal.pone.0050027 (PMC3521728; doi:10.1371/journal.pone.0050027)
Supplement: Table S3 — Analytical characterization of aS dimers fragments obtained by proteolysis with proteinase K of aggregated species, after removal of the soluble species by ultracentrifugation. (PDF) [file pone.0050027.s003.pdf]

Table S3. Analytical characterization of aS dimers fragments obtained by proteolysis with proteinase K of aggregated species, after removal of the soluble species by ultracentrifugation.

Protein	RT[a]	Experimental Mass (Da)[b]	Calculated Mass (Da)[c]	Fragment[d]
aS	27.8	5663.25 ±0.07	5662.62	1-56
	28.8	8815.5 ±0.41	8815.58	57-140
		7058.85 ±0.25	7059.79	57-125
	30.2	11411.98 ±0.78	11412.59	31-140
		12596.29 ±0.78	12596.1	19-140
	30.5	9655.9 ±0.07	9656.8	31-125
	33.1	14458.8 ±0.1	14460.19	1-140
	33.6	12702.8 ±0.67	12704.39	1-125
	34.4	11330.01 ±0.04	11327.97	1-113
NN	13.3	3798.41 ±0.21	3728.26	19-56*
		3870.37 ±0.33	3870.42	18-56*
	28.8	8816.21 ±0.19	8815.58	57-140*
		7059.69 ±0.15	7059.79	57-125*
	30.1	12597.42 ±0.56	12596.1	19-140*
		10841.56 ±0.74	10841.12	19-125*
	30.6	9464.32 ±0.85	9464.7	19-113*
CC	28.2	10928.52 ±0.12	10932.86	57-142ss126-142
	28.7	7059.8 ±0.04	7059.79	57-125*
		5683.24 ±0.09	5683.37	57-113*
	28.9	14671.84 ±0.54	14673.09	19-142ss126-142
		14603.49 ±0.27	14602.02	20-142ss126-142
	29.9	10842.19 ±0.06	10841.12	19-125*
		10770.97 ±0.02	10770.05	20-125*
		10912.83 ±0.25	10912.19	18-125*
	30.5	9465.42±0.15	9464.7	19-113*
		9394.14±0.15	9393.62	20-113*
		9536.59 ±0.05	9535.78	18-113*
	32.1	16555.24 ±0.35	16540.38	1-142ss126-142
	32.3	12706.82 ±0.12	12704.4	1-125*
	33.8	11330.69 ±0.98	11327.98	1-113*
	34.8	29225.24 ±1.02	29226.76	1-142ss1-142
NC	13.1	3799.22±0.15	3799.26	19-56*
		3728.19±0.15	3728.26	20-56*
		2614.58±0.16	2615.03	31-56*
	27.9	5663.25 ±0.67	5662.62	1-56*
	28.8	7059.21 ±0.55	7059.79	57-125*
		8815.5 ±0.07	8815.58	57-140*
	30.2	12596.24 ±0.22	12596.1	19-140*
	30.5	10771.33 ±0.24	10770.05	19-125*
		10840.24 ±0.24	10841.12	20-125*
		12752.41 ±0.32	12753.1	19-141
	33.5	14615.83 ±0.33	14616.37	1-141
		14934.1 ±0.27	14934.65	1-143
	34.1	12704.27 ±0.43	12704.4	1-125*

DC[e]	29.5	8818.04 ±0.10	8818.09	32-104/29-43
		7059.69 ±0.04	7059.79	57-125
		5684.30 ±0.39	5684.45	57-113
		6063.90 ±1.58	6062.3	57-104/29-41
		5744.26±0.15	5743.51	57-104/29-38
	29.9	6086.68±0.15	6085.91	53-104/29-38
		9157.64 ±0.24	9157.98	53-140
		7402.35 ±0.10	7402.18	53-125
	30.6	10280.90 ±0.76	10280.26	42-140
		10192.89 ±0.75	10193.19	43-140
		7211.06 ±0.07	7211.2	45-104/29-41
		7527.40 ±0.11	7527.56	42-104/29-41
		8437.18 ±0.21	8437.4	43-125
		8526.36 ±0.21	8524.48	42-125
	30.9	8660.06 ±0.48	8659.88	31-104/29-41
		7059.89 ±0.29	7059.79	57-125
		7048.37 ±0.59	7148.06	42-113
		8342.67 ±0.34	8340.56	31-104/29-38
		16325.85 ±0.56	16325.13	57-104/29-140
	32.2	6933.11 ±0.41	6932.81	44-113
		7470.42 ±0.16	7470.51	52-104/29-50
		14570.46 ±0.76	14570.53	43-104/29-113
		20035.01 ±0.57	20035.38	20-104/29-140
		20106.02 ±0.38	20106.45	19-104/29-140
	35.2	18922.64 ±0.58	18922.14	31-104/29-140
		17789.17 ±0.22	17789.81	42-104/29-140
		21969.71 ±0.67	21969.73	1-104/29-140
		20213.48 ±0.54	20213.94	1-104/29-125

[a] The peptides obtained from the proteolysis of aS and its dimers with proteinase K were purified by RP-HPLC and listed in order of retention times (RT).

[b] Experimental molecular masses determined by ESI-MS, using a Q-Tof instrument (Waters, Milford).

[c] Molecular masses calculated from the amino acid sequence.

[d] Peptides that can belong to both of the aS molecules that constitute the dimer are indicated by a star (*).The disulphide bridge between the two molecule constituting NN and CC dimers is indicated by ss.

[e] As DC is constituted by the sequence 1-104 linked to 29-140 of aS, the sequence notation of its proteolytic fragments refers to the aS sequence numbering. In the case of fragments encompassing the two sequences, these were indicated with numbering of the segments deriving from both sequences separated by a slash.
