# Supplementary material for: Covalent α-Synuclein Dimers: Chemico-Physical and Aggregation Properties
Source: PLoS One. 2012 Dec 13;7(12):e50027. doi: 10.1371/journal.pone.0050027 (PMC3521728; doi:10.1371/journal.pone.0050027)
Supplement: Table S4 — Analytical characterization of aS dimers fragments obtained by proteolysis with trypsin of aggregated species, after removal of the soluble species by ultracentrifugation. (PDF) [file pone.0050027.s004.pdf]

Table S4. Analytical characterization of aS dimers fragments obtained by proteolysis with trypsin of aggregated species, after removal of the soluble species by ultracentrifugation.

Protein	RT[a]	Experimental Mass (Da)[b]	Calculated Mass (Da)[c]	Fragment[d]
aS	30.2	12039.3 ±0.4	12039.26	24-140
		11227.51 ±0.27	11227.37	33-140
		13123.54 ±0.51	13123.5	13-140
		13322.94 ±0.54	13322.75	11-140
	30.8	10998.39 ±0.07	10998.09	35-140
		6956.44 ±0.41	6956.96	33-102
	31.2	6727.32 ±0.31	6727.68	35-102
		8552.39 ±0.84	8552.62	35-119
		6185.27 ±0.69	6186.07	35-97
		6414.93 ±0.64	6415.35	33-97
	32.7	14460.53 ±0.22	14460.19	1-140
	33.6	6057.28 ±0.04	6057.9	35-96
NN	30.3	12039.18 ±0.93	12039.26	1-24ss1-140
		10994.53±0.64	10993.82	1-12ss1-96
		13798.01 ±1.58	13795.02	1-99ss1-96
	30.9	12074.05 ±0.93	12076.46	1-22ss1-96
CC	32	19068.49 ±0.76	19067.85	44-142ss58-142
	36	29226.37 ±0.9	29226.76	1-142ss1-142
NC	30.2	12038.77 ±0.31	12039.26	24-140*
		12195.01 ±0.32	12195.45	24-141
	32.7	14616.66 ±0.32	14616.37	1-141
	33.7	1477.85 ±0.1	1477.78	81-96*
DC[e]	28	3464.1 ±0.3	3434.32	46-80*
		3664.99 ±0.11	3663.4	44-80*
	28.5	4596.75 ±0.38	4597.24	35-80*
		4826.11 ±0.45	4856.55	33-80*
		5638.29 ±0.30	5638.46	24-80*
	29.7	4858.40 ±0.37	4858.49	59-104/29-32
		4288.38 ±0.38	4288.43	103-140
	30.6	4058.22 ±0.02	4058.63	61-103
	31.9	10065.83 ±0.24	10065.01	44-140
	33.1	10998.95 ±0.72	10997.49	35-140
		5565.09 ±0.46	5564.75	46-103
		5125.95 ±0.02	5124.23	44-97
	37.8	19548.8 ±0.38	19548.8	24-104/29-140

[a] The peptides obtained from the proteolysis of aS and its dimers with proteinase K were purified by RP-HPLC and listed in order of retention times (RT).

[b] Experimental molecular masses determined by ESI-MS, using a Q-Tof instrument (Waters, Milford).

[c] Molecular masses calculated from the amino acid sequence.

[d] Peptides that can belong to both of the aS molecules that constitute the dimer are indicated by a star (*).The disulphide bridge between the two molecule constituting NN and CC dimers is indicated by ss.

[e]As DC is constituted by the sequence 1-104 linked to 29-140 of aS, the sequence notation of its proteolytic fragments refers to the aS sequence numbering. In the case of fragments encompassing the

two sequences, these were indicated with numbering of the segments deriving from both sequences separated by a slash.
