# Supplementary figures and images for: Covalent α-Synuclein Dimers: Chemico-Physical and Aggregation Properties
Source: PLoS One. 2012 Dec 13;7(12):e50027. doi: 10.1371/journal.pone.0050027 (PMC3521728; doi:10.1371/journal.pone.0050027)

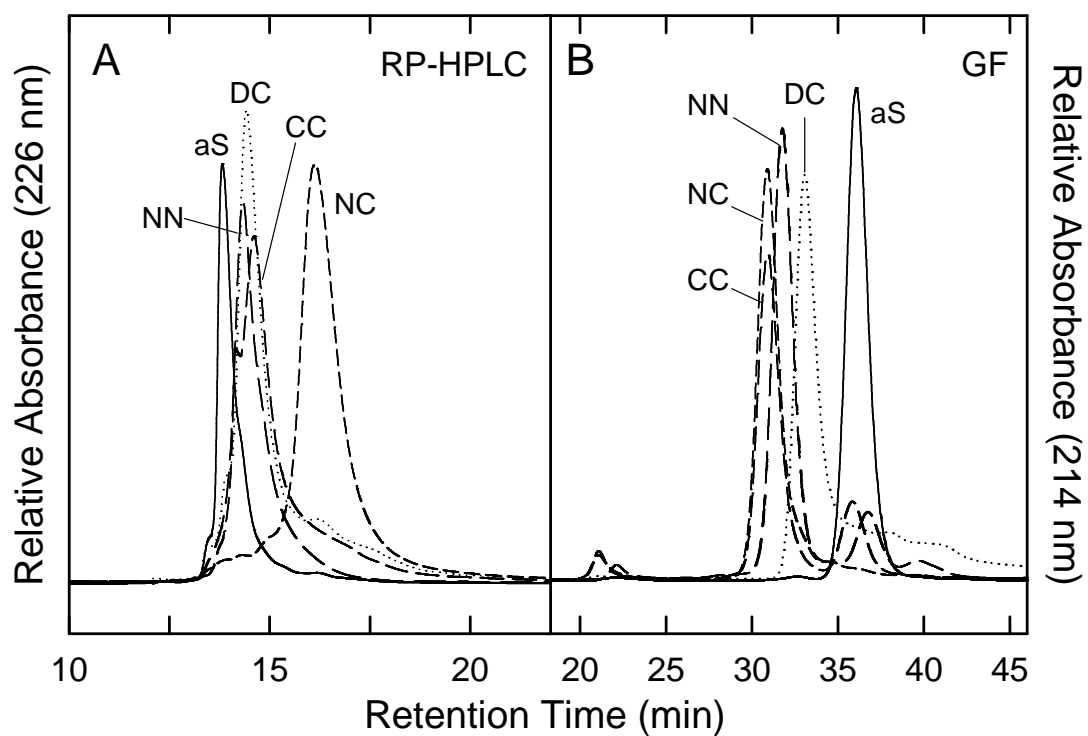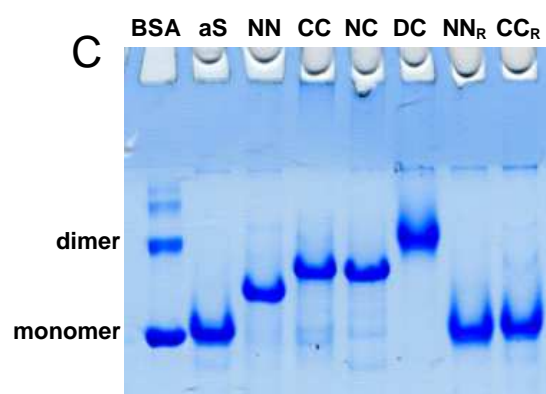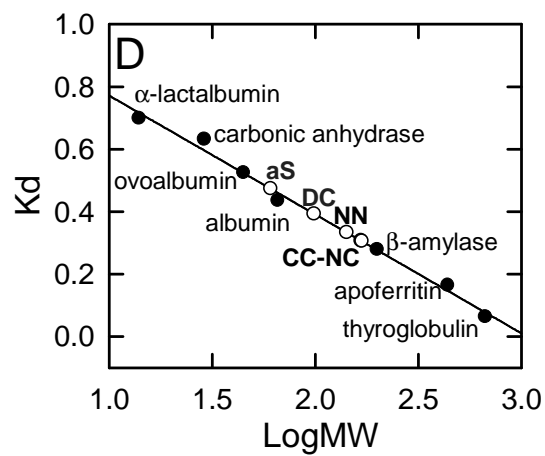

Supplement: Figure S1 — Chemical characterization of aS dimers by RP-HPLC (A), gel filtration (GF) chromatography (B) and native gel electrophoresis (C). RP-HPLC was conducted on C4 column using a liner gradient of acetonitrile. GF experiments were conducted with a Superdex 200 column, eluted with Tris-HCl buffer (20 mM Tris, 150 mM NaCl, pH 7.4) at flow rate of 0.4 ml/min. aS, NN, CC, NC and DC are represented respectively by a continuous, long dash, medium dash, short dash and dotted line. GF calibration (D) was obtained using α-lactalbumin, carbonic anhydrase, ovalbumin, bovine serum albumin (BSA), β-amylase, apoferritin and thyroglobulin, as protein reference molecular markers (black dots). Proteins hydrodynamic volumes were calculated on the basis of their Kd (white dots). In the native PAGE, BSA was used as marker (first lane); NNR and CCR represent disulfide-bond containing dimers after reduction by β-mercaptoethanol. (PDF) [file pone.0050027.s005.pdf]

Relative Absorbance

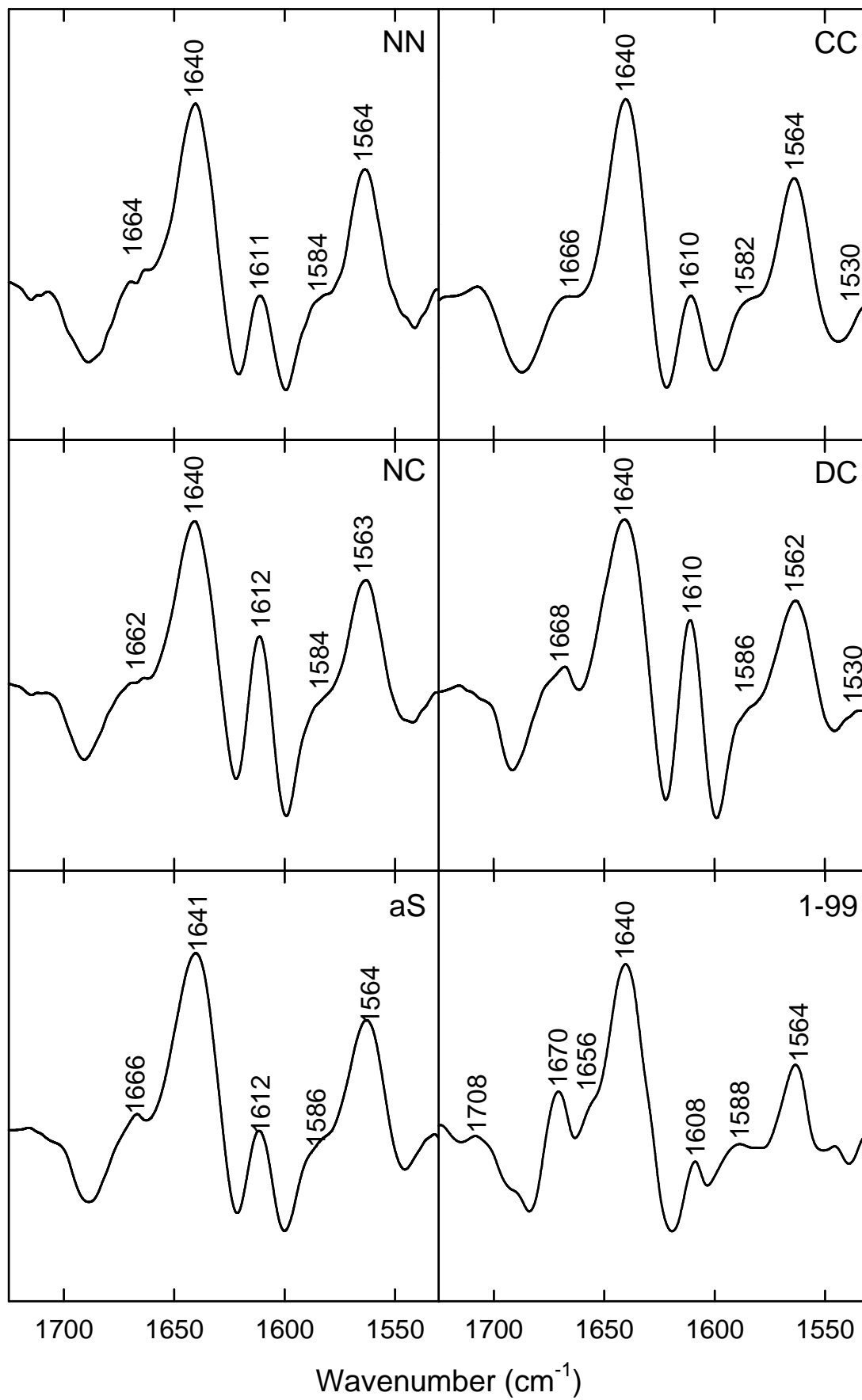

Supplement: Figure S2 — Second derivative of FT-IR spectra of aS dimers and 1–99 of the spectra reported in Fig. 2 . The relative values of the bands were used as reference for the curve fitting operation. (PDF) [file pone.0050027.s006.pdf]

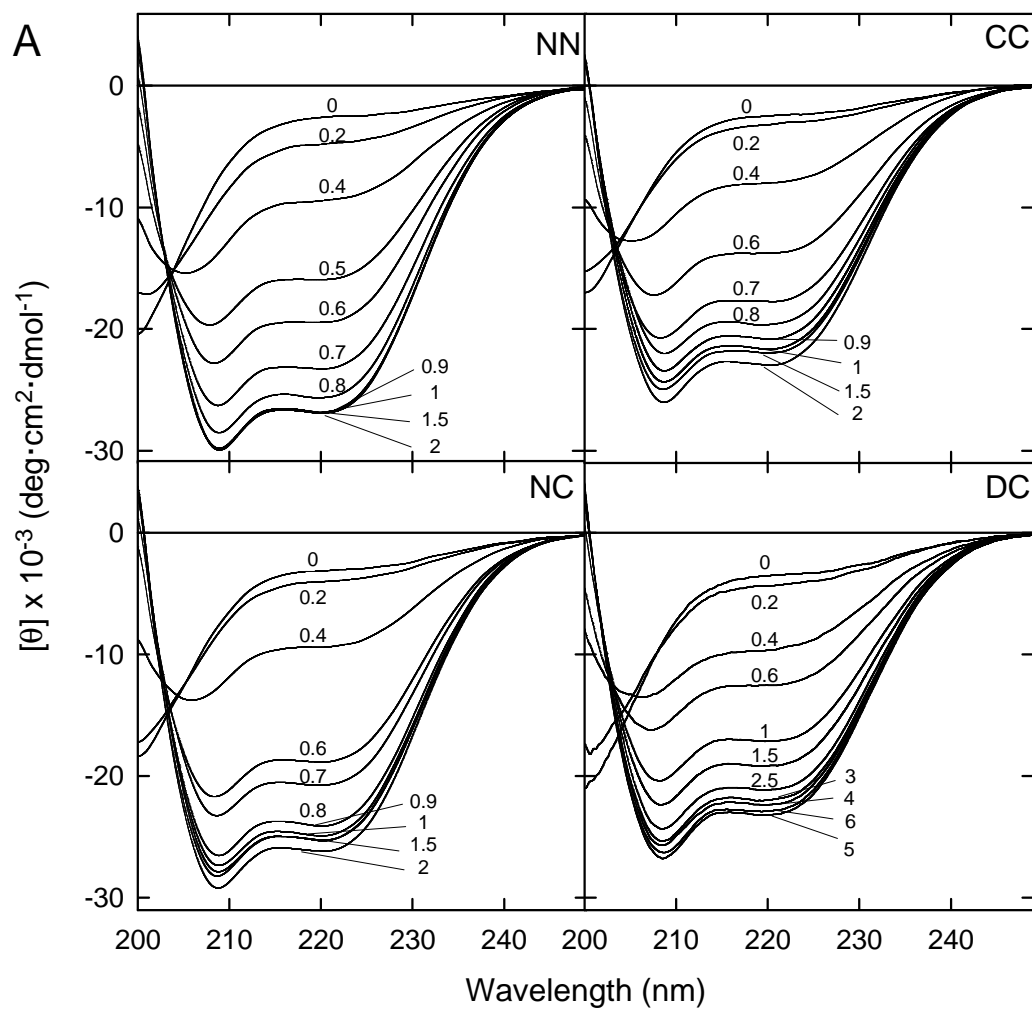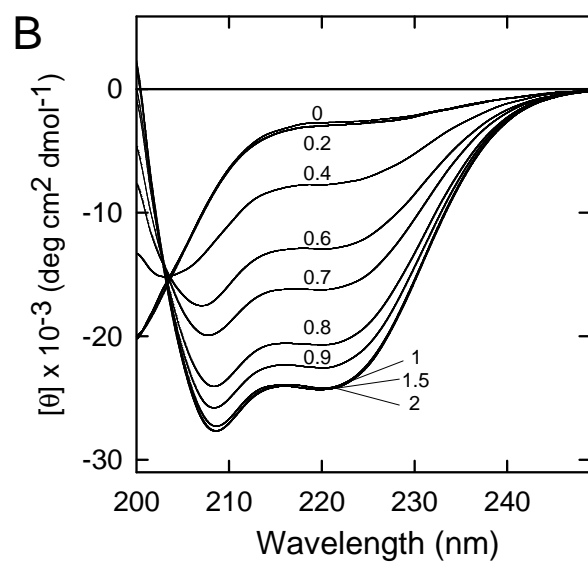

Supplement: Figure S3 — Far UV CD spectra of aS dimers (A) and aS (B) in the presence of increasing concentration of SDS. The spectra were recorded in PBS buffer pH 7.4 at a protein concentration of 20 µM. The numbers close to the curves indicate SDS concentrations (mM). (PDF) [file pone.0050027.s007.pdf]

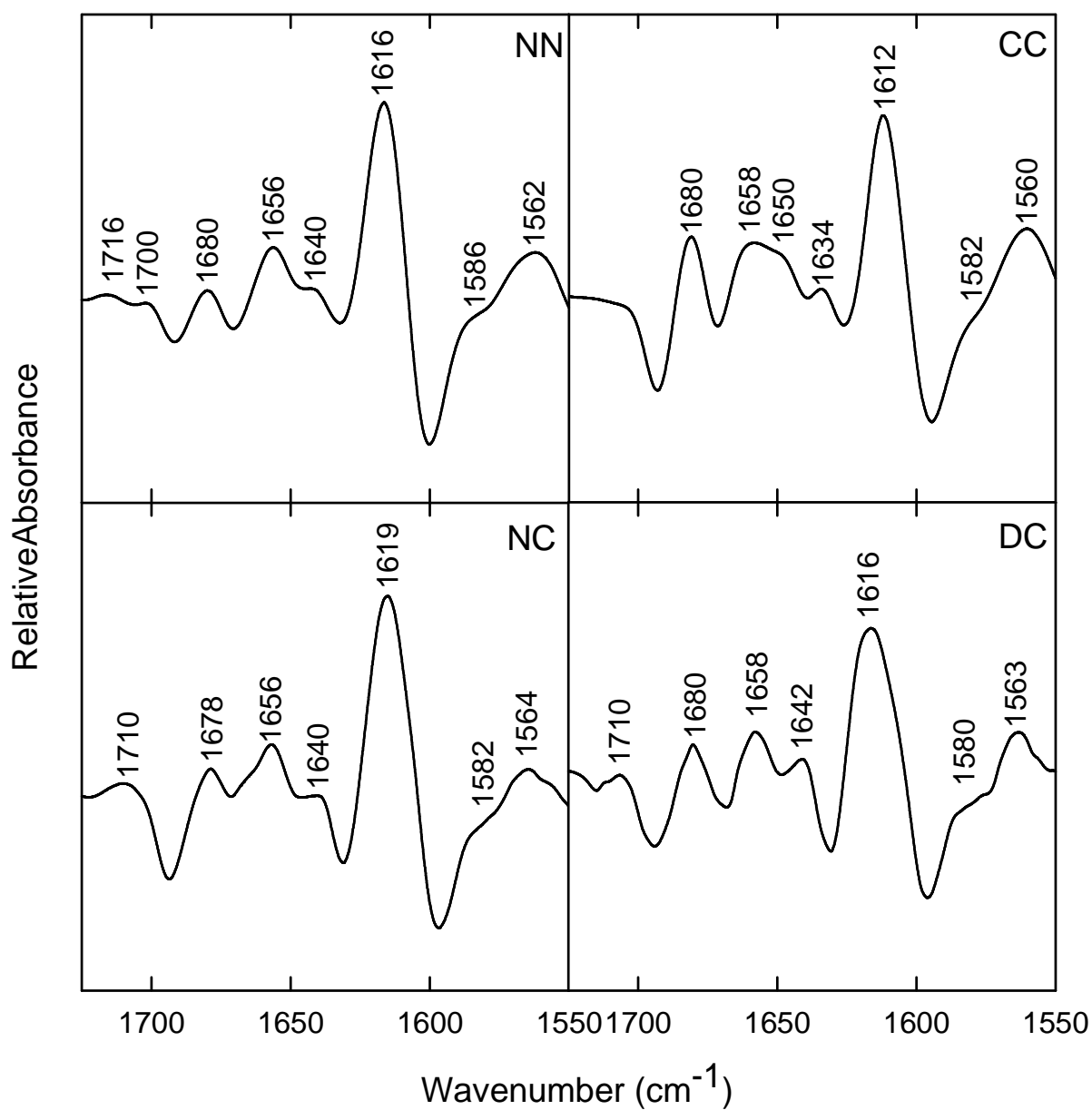

Supplement: Figure S4 — Second derivative of FT-IR spectra of aS dimers aggregates reported in Fig. 6 . (PDF) [file pone.0050027.s008.pdf]

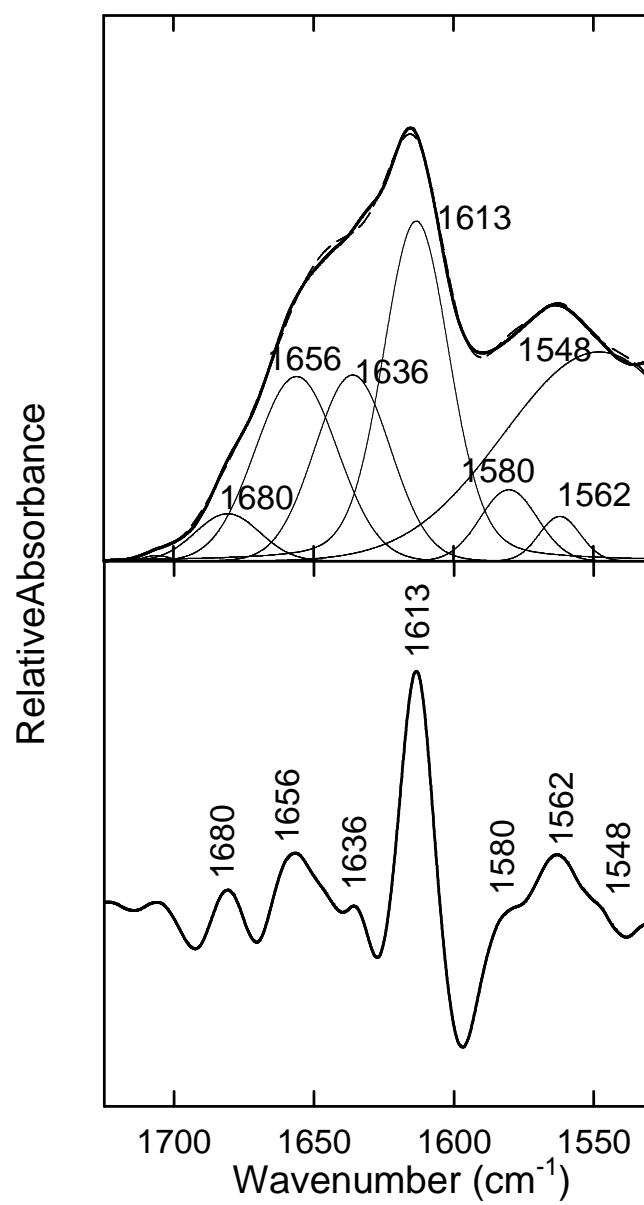

Supplement: Figure S5 — FT-IR spectra of aS fibrils obtained after one month of protein incubation. The peak positions of the amide band components were deduced from the second derivative spectra (lower panel). The sum of the fitted curves is shown as a broken line, closely overlapping the experimental trace, shown as a continuous line. (PDF) [file pone.0050027.s009.pdf]

aS

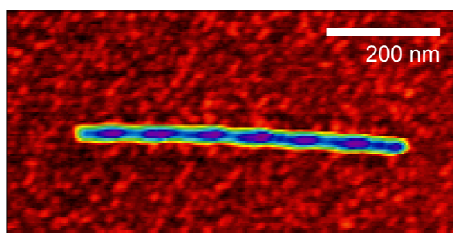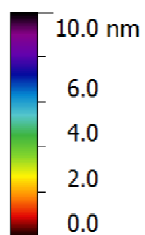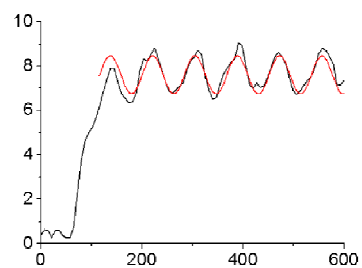

NC

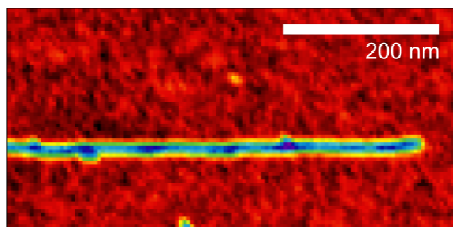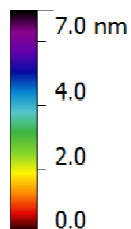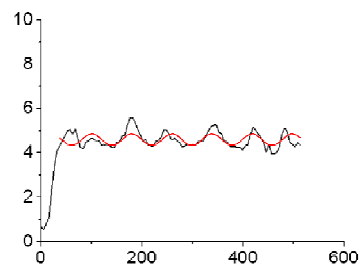

NN

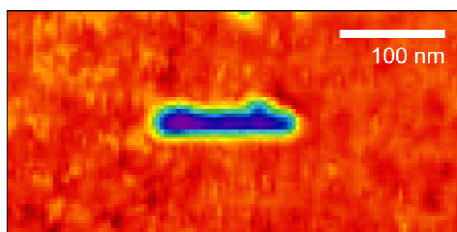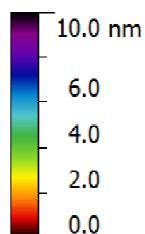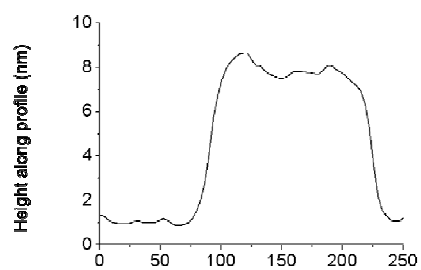

CC

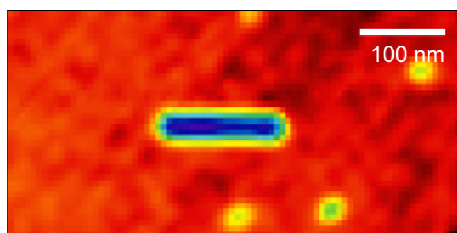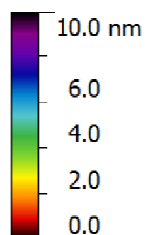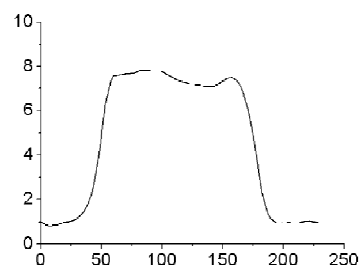

DC

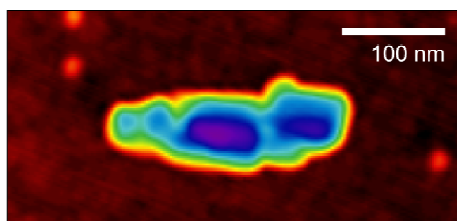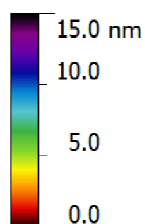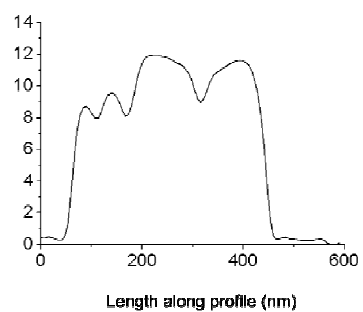

Supplement: Figure S6 — AFM images (left column) and height profiles along horizontal sections (right column) of representative individual fibrillar aggregates composed by each of aS dimers used in this study. aS and NC height profiles are superimposed with a sinusoidal fit (solid red line). The best fitting sinusoid periods are 84 nm and 79 nm for aS and NC respectively. (PDF) [file pone.0050027.s010.pdf]

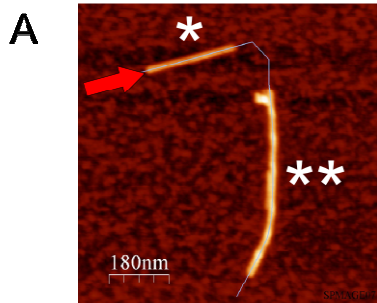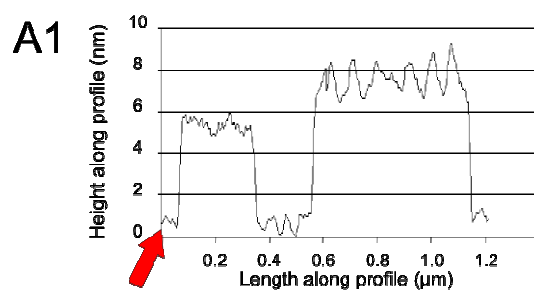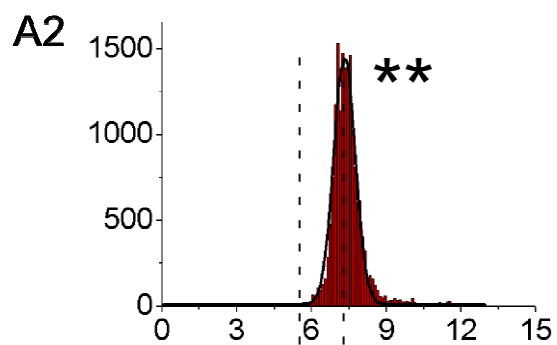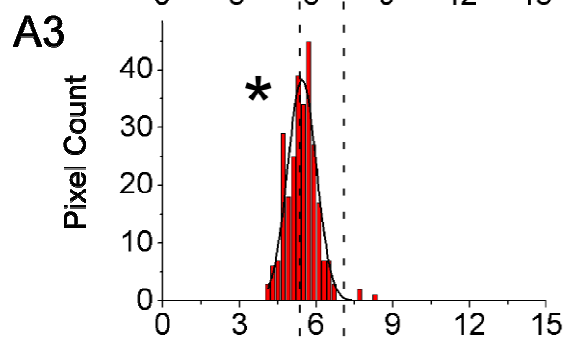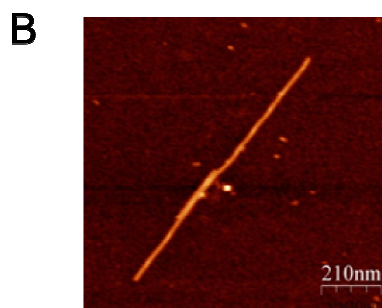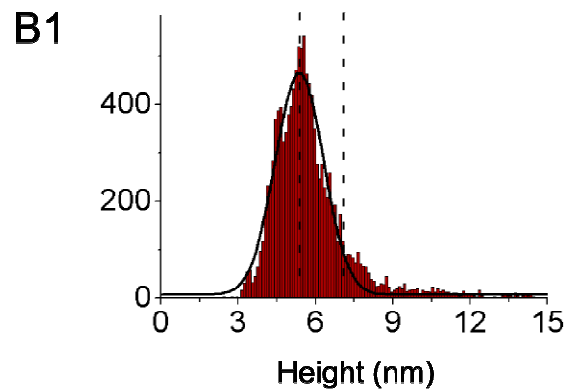

Supplement: Figure S7 — (A) AFM image showing two different types of aS fibrillar aggregates. A rarely observed protofibril (*) lies near a mature fibril (**). (B) AFM image of representative NC dimer aggregates. (A1) Height profile of the fibrillar aggregates shown in panel A, measured along a broken line section (pale blue solid line in panel A). (A2, A3, B1) Distribution of all the measured diameters of mature aS fibrils (A2, **) aS protofibrils (A3, *), and NC fibrils (B1). Gauss fits are superimposed to each distribution (solid black lines) and are centered respectively on 7.3 nm (aS mature fibrils), 5.4 nm (aS protofibrils) and 5.4 nm (NC fibrils). (PDF) [file pone.0050027.s011.pdf]
